# Dose- and modality-specific effects of exercise on BDNF and depression in middle-aged and older adults with mild cognitive impairment: A Bayesian network meta-analysis

**DOI:** 10.1371/journal.pone.0358178

**Published:** 2026-10-07

**Authors:** Gong Cheng, Jinhui Qiu, Shengtao Li

**Affiliations:** 1 Hoseo University, Asan-si, Republic of Korea; 2 The College of Physical Education, Minnan Normal University, Zhangzhou, Fujian, China; Northeastern University, CHINA

## Abstract

**Objective:**

To evaluate the effects of exercise modality and weekly exercise dose on peripheral brain-derived neurotrophic factor (BDNF) and depressive symptoms in middle-aged and older adults with mild cognitive impairment (MCI), and to examine the robustness of the dose-specific predictions from the primary quadratic model across alternative dose–response functions.

**Methods:**

PubMed, Embase, Web of Science, the Cochrane Library, and APA PsycInfo were searched from inception to 13 April 2026. Randomized controlled trials involving adults aged 50 years or older with MCI and reporting BDNF or depression-related outcomes were included. Effect sizes were expressed as Hedges’ g. Separate Bayesian random-effects network dose–response meta-analyses were conducted for BDNF, overall depressive symptoms, and modality-specific depressive symptoms. The quadratic model was used as the primary model to predict intervention effects at different exercise doses. Linear, Emax, and natural cubic spline models were fitted as functional-form sensitivity analyses.

**Results:**

Twenty-eight studies involving 2,262 participants were included. In the primary quadratic analysis, the largest model-predicted effect on BDNF occurred at 440 MET-min/week (posterior mean, 0.752; 95% credible interval [CrI], 0.287 to 1.249), whereas the largest model-predicted effect on depressive symptoms occurred at 670 MET-min/week (posterior mean, 0.382; 95% CrI, 0.147 to 0.674). Dancing and multicomponent exercise showed comparatively favourable estimates at selected dose levels, whereas estimates for Tai Chi, walking, and yoga were less precise. Functional-form sensitivity analyses identified different dose locations for the maximum predicted effects. Therefore, 440 and 670 MET-min/week represent the maximum prediction points for BDNF and depressive symptoms, respectively, under the primary quadratic model, rather than established optimal exercise doses.

**Conclusion:**

Exercise interventions were associated with increased peripheral BDNF levels and improved depressive symptoms in middle-aged and older adults with MCI. The primary quadratic model predicted the largest effects at 440 MET-min/week for BDNF and 670 MET-min/week for depressive symptoms, suggesting a potentially non-linear dose–response relationship. Although these prediction points are important findings of the quadratic analysis, cross-model sensitivity indicates that they should be used to inform future dose-ranging research rather than be regarded as confirmed clinical optima.

## 1. Introduction

Mild cognitive impairment (MCI) is a clinical syndrome characterized by cognitive decline greater than expected for age and education, while daily functional independence is largely preserved and diagnostic criteria for dementia are not met [1,2]. It is therefore distinct from both dementia and broadly defined cognitive impairment. Epidemiological studies indicate that MCI is common among middle-aged and older adults and is associated with an elevated risk of progression to dementia, although conversion rates vary by setting, diagnostic criteria, follow-up duration, and baseline clinical characteristics [1,3,4].

Management of MCI commonly emphasizes risk-factor control, cognitive and lifestyle interventions, and monitoring of progression. At present, no broadly applicable disease-specific pharmacological treatment is recommended for MCI as a syndrome, although disease-modifying therapies may be relevant to selected individuals with biomarker-confirmed early Alzheimer’s disease [5–7]. Exercise has therefore received attention as a potentially scalable non-pharmacological strategy. However, previous exercise reviews in MCI have focused mainly on global cognitive outcomes, whereas depressive symptoms and biological markers related to neuroplasticity, particularly brain-derived neurotrophic factor (BDNF), have received less integrated attention [8,9].

Exercise may influence depressive symptoms in individuals with MCI through both psychosocial and neurobiological pathways. Psychosocially, physical activity may reduce depressive and anxiety symptoms by improving self-efficacy, self-concept, and self-esteem, whereas neurobiologically, exercise-related changes may involve neuroplasticity-related biomarkers and brain structural or functional adaptations [10,11]. BDNF is a key neurotrophin involved in synaptic development, synaptic plasticity, neuronal survival, and activity-dependent adaptation, and it has also been implicated in psychiatric disorders and mood regulation [12,13]. Therefore, examining BDNF and depressive symptoms together may provide a theoretically meaningful framework for understanding the potential psychobiological effects of exercise in middle-aged and older adults with MCI [14].

Accordingly, this study focused on middle-aged and older adults aged 50 years [15] or above with MCI and used a Bayesian model-based network dose–response meta-analysis to synthesize evidence across exercise modalities and weekly dose levels. This approach was selected because it can combine direct and indirect comparisons and allows exploratory modelling of non-linear dose–response patterns when interventions differ in both modality and dose. The theoretical contribution of this study is to clarify whether BDNF levels and depressive symptoms show similar or divergent dose-related patterns in MCI. The practical contribution is to identify dose ranges and exercise modalities that warrant further testing in future trials, rather than to establish definitive clinical prescriptions based on the current evidence alone.

## 2. Methods

### 2.1. Data sources and search strategy

This study was conducted as a systematic review and meta-analysis to synthesise the available evidence in a structured manner. The design, implementation and reporting of the review followed the Preferred Reporting Items for Systematic Reviews and Meta-Analyses (PRISMA) guidelines. To enhance transparency and procedural rigour, the study protocol was prospectively registered in the PROSPERO database under registration number CRD420261371703.

The electronic database searches were last updated on 13 April 2026. Supplementary searching was limited to manual screening of the reference lists of included studies and relevant systematic reviews; no trial registry, dissertation database, conference database, or other grey-literature source was searched. The search strategy combined controlled vocabulary and free-text terms for MCI, exercise or physical activity, BDNF, and depressive symptoms. The manual search identified 43 records. Before screening, 39 were removed because they were duplicates or had already been identified in the electronic database searches. Four unique manual records were screened, two were excluded during title/abstract screening, and two were assessed in full text; both full-text reports were excluded, so no manually identified study was added to the final set. All records were managed in the same screening workflow. Two reviewers independently screened records, with disagreement resolved by discussion or a third reviewer. Full database strategies are provided in S1 Table (S1 File).

### 2.2. Eligibility criteria

Eligibility criteria were established according to the PICOS framework and were defined across five domains: participant characteristics, intervention content, comparator type, outcome measures and study design.

Eligible participants were middle-aged and older adults aged 50 years or older with MCI diagnosed using clearly stated standardized criteria, such as Petersen criteria, National Institute on Aging–Alzheimer’s Association criteria, Montreal Cognitive Assessment-based criteria, Mini-Mental State Examination-based criteria, or other validated clinical diagnostic approaches. The age threshold of 50 years was used to capture both middle-aged and older adults at risk of later-life cognitive decline while reducing heterogeneity from younger-onset neurological conditions. Studies including dementia, Parkinson’s disease, stroke, major psychiatric disorders, or other central nervous system disorders were excluded unless data for the MCI subgroup could be extracted separately.

Eligible interventions were required to be centred on exercise or physical activity. These included aerobic exercise, resistance exercise, multicomponent training, and combined programmes integrating exercise with virtual reality, cognitive tasks, or game-based interaction. Complex interventions were included only when exercise or physical activity was the core component of the intervention. Interventions combined with virtual reality, cognitive tasks, or game-based activities were coded according to their dominant exercise component and delivery format. When non-exercise components were present, this information was extracted and considered in the assessment of transitivity and in the interpretation of modality-specific findings. Because the subsequent analyses aimed to compare the dose composition of different exercise programmes, no uniform restrictions were imposed at the screening stage on training frequency, intensity, session duration, total intervention period, or rest arrangement.

Eligible comparators included inactive, non-exercise, and active comparator conditions, such as usual care, conventional rehabilitation, traditional physical therapy, routine activity guidance, non-exercise cognitive training, stretching, or another exercise intervention. Because this study used a network meta-analytic framework, trials directly comparing different exercise modalities or dose levels were also eligible and contributed to direct and indirect comparisons across the evidence network, provided that the intervention arms were clearly defined and distinguishable. Comparator type was extracted and considered when assessing clinical and methodological transitivity.

Studies were eligible if they reported extractable data for either BDNF or depression-related outcomes. Because BDNF and depressive symptoms were analysed as two separate endpoints rather than as a combined outcome, studies were not required to report both outcomes. Accordingly, the BDNF and depression networks were constructed separately using the studies that provided extractable data for each specific endpoint. Only randomized controlled trials published as full-text articles in peer-reviewed journals were eligible. Trial registries and grey-literature sources were not searched, and unpublished records were therefore not considered for eligibility or quantitative synthesis. Study protocols, conference abstracts, dissertations, observational studies, case series, and non-randomized studies were excluded.

### 2.3. Data processing and extraction

To improve the objectivity and reproducibility of data handling, study screening and data extraction were conducted independently by two reviewers using a predefined data extraction form. The process included management of search records, title and abstract screening, full-text eligibility assessment, and extraction of study, participant, intervention, comparator, outcome, and statistical information. Discrepancies during study selection or data extraction were resolved by rechecking the original articles and discussion between the two reviewers. If disagreement persisted, a third reviewer was consulted to make the final decision. Reviewer agreement was checked during the screening and extraction process before consensus decisions were made.

Four categories of information were extracted. First, study background characteristics were recorded, including first author, year of publication, country or region, exercise timing, and study design. Second, participant characteristics were collected, including sample size, age, and sex distribution. Third, exercise prescription characteristics were extracted, including exercise programme, intervention modality, weekly frequency, total intervention period, session duration, and assigned metabolic equivalent value. Fourth, outcome-related information was recorded, including whether the study reported BDNF, depression-related outcomes, or both, as well as the statistical data required for effect size estimation.

Effect sizes were calculated as Hedges’ g. When post-intervention means and standard deviations were available, effect sizes were based on post-intervention between-group differences. When only change scores were available, change-score differences were used. If standard deviations of change scores were not reported, they were estimated using an assumed pre–post correlation coefficient of 0.50. For multi-arm trials sharing one control group, the control-group sample size was divided across relevant comparisons to avoid double counting. When multiple follow-up time points were reported, the assessment immediately after completion of the intervention was used as the primary endpoint. If multiple depression scales were reported in the same study, only one scale was selected for analysis [16]. Missing or incomplete outcome data were handled according to predefined rules. When means and standard deviations were not directly reported, available statistics such as standard errors, confidence intervals, P values, medians, ranges, interquartile ranges, and sample sizes were converted to the required format using recommended methods from the Cochrane Handbook. When change-score standard deviations were unavailable, they were estimated using an assumed pre–post correlation coefficient of 0.50. No imputation was performed for missing individual participant data, because this review was based only on aggregate data extracted from published studies. If the required data could not be obtained or converted into a form suitable for effect size estimation, the study was excluded from the corresponding quantitative analysis for that specific outcome. The predefined rules for effect-size calculation and data handling are provided in S2 File.

### 2.4. Methodological quality assessment

As all included evidence was derived from randomized controlled trials, the methodological quality of the eligible studies was assessed using the Revised Cochrane risk-of-bias tool for randomized trials (RoB 2.0) [17]. The use of an appraisal tool aligned with the study design allowed more targeted identification of potential sources of bias arising during the conduct and reporting of randomized studies and provided a methodological basis for judging the overall reliability of the evidence.

Risk of bias was assessed independently by two trained reviewers using RoB 2.0. Disagreements were resolved through re-examination of the original article and discussion between the reviewers. If consensus could not be reached, a third reviewer undertook an additional assessment and made the final decision. RoB 2.0 evaluates bias arising from the randomization process, deviations from intended interventions, missing outcome data, outcome measurement, and selection of the reported result [17]. In the present study, risk-of-bias judgments were used to describe the methodological quality of the included studies and to guide the interpretation of findings and GRADE certainty ratings rather than to exclude studies from the primary analysis.

### 2.5. Model construction and analytical procedures

A Bayesian random-effects model-based network dose–response meta-analysis was performed to evaluate associations between weekly exercise dose and BDNF or depressive outcomes in patients with mild cognitive impairment. Before model fitting, network geometry was examined to determine whether the intervention nodes formed a connected evidence network. To assess transitivity, available clinical and methodological characteristics were reviewed across the included studies, including age, sex distribution, sample size, exercise programme, intervention duration, training frequency, session duration, assigned MET value, reported outcome, risk of bias, and comparator condition. Based on the available information, no clear systematic imbalance in these characteristics was identified across the main treatment nodes. However, MCI diagnostic criteria, baseline depression severity, and several other potential effect modifiers were not consistently reported in all studies. Therefore, although the available evidence did not indicate an obvious violation of the transitivity assumption, transitivity could not be fully verified. Local inconsistency was examined using node-splitting analyses. Because node-splitting evaluates disagreement between direct and indirect evidence rather than transitivity itself, and may have limited power for comparisons supported by limited evidence, non-significant results were interpreted cautiously rather than as definitive proof that all network assumptions were satisfied [18,19].

Effect sizes were expressed as standardized mean differences with Hedges’ g correction. Separate Bayesian random-effects dose–response networks were fitted for overall exercise dose–BDNF and overall exercise dose–depressive symptom relationships [20]. Four candidate functions were evaluated within each overall outcome network: linear, quadratic polynomial, Emax, and natural cubic spline, with three internal knots placed at the 0.1, 0.5, and 0.9 quantiles of the observed dose distribution [21,22]. The modality-specific depressive symptom network used a quadratic descriptive function and was restricted to dancing, multicomponent exercise, Tai Chi, walking, and yoga because each modality had at least two observed dose levels.

All models used a normal likelihood, a standardized mean difference link, and a random-effects structure. The deviance information criterion (DIC) was used to compare candidate-model fit within each outcome network and was not compared across different outcome networks. Predictions were expressed relative to placebo (E0 = 0) and restricted to the observed dose range of 0–1,000 MET-min/week. Posterior mean dose–response curves from the candidate models were overlaid for visual comparison. For each retained posterior draw, the dose associated with the maximum predicted effect was identified using a 10-MET-min/week grid, thereby generating the posterior distribution of the maximizing dose.

All models were run using three Markov chains with 20,000 iterations per chain, of which the first 10,000 were discarded as burn-in. A thinning interval of 10 was applied, yielding 3,000 retained posterior draws per model. Convergence was prespecified as a maximum Gelman–Rubin R-hat of ≤1.05 and a minimum effective sample size (ESS) of ≥400 for the monitored stochastic parameters. Study-specific baseline parameters and dose–response coefficients were assigned Normal(0, precision = 0.0001) priors. The ED50 parameter in the Emax model was assigned a Normal(0, 0.0001) prior truncated below at 0. The between-study heterogeneity standard deviation was assigned a Uniform(0, 1.667) prior for the BDNF network and a Uniform(0, 1.419) prior for both depressive symptom networks. Analyses were conducted in R and JAGS using MBNMAdose. Complete code, exact model-specific priors, initialization settings, software versions, and session information are provided in S2 Files.

Weekly exercise dose was calculated for each intervention arm as the assigned MET value × minutes per session × sessions per week and was expressed as METs·min/week [23]. Because most original trials did not directly report MET values, MET values were derived from the 2024 Adult Compendium of Physical Activities by matching the exercise modality and intensity described in each trial report to the most closely corresponding Compendium activity code [24]. When an intervention included multiple exercise components and the duration of each component was available, a time-weighted mean MET value was calculated according to the duration of the respective components. When component-specific durations were unavailable, the reported intervention characteristics were reviewed in detail, and the Compendium activity code that most closely corresponded to the dominant exercise component and the intensity described by the original investigators was selected. Because these assignments relied on less detailed intervention information, they were identified as having greater uncertainty.

The calculated continuous weekly exercise doses were retained separately from the categorical dose nodes used for network construction. For network construction and node-splitting analyses, each intervention arm was assigned to the nearest dose node at 250-METs·min/week intervals. This interval was selected to preserve meaningful differentiation between the observed exercise doses while avoiding excessive fragmentation of the available evidence into sparsely populated or disconnected nodes, thereby balancing dose resolution, network connectivity, and the stability of indirect comparisons. The original continuous dose values, rather than the categorized node values, were used for model-based dose–response estimation to minimize information loss resulting from categorization. Model-predicted dose–response estimates were generated only across the observed dose range. Study-level intervention descriptions, assigned MET values, intervention duration, training frequency, session duration, and calculated weekly exercise doses are provided in S3 File. The dose-conversion and node-construction rules, together with the model diagnostic materials, are summarized in S2 File [25].

### 2.6. Evidence grading and assessment of result robustness

To strengthen interpretation of the credibility of the main findings, the quality of evidence was evaluated separately for each core outcome. These assessments were conducted independently by two reviewers. If differences in grading arose, the original studies were re-examined according to prespecified criteria and cross-checked again. If disagreement persisted, a third reviewer was consulted to adjudicate and determine the final rating.

The certainty of evidence was assessed using the GRADE framework across risk of bias, inconsistency, indirectness, imprecision, and publication bias. Publication-bias judgments were informed by contour-enhanced funnel plots, Egger’s regression test, trim-and-fill analyses, and descriptive sunset funnel plots. Evidence certainty was classified as high, moderate, low, or very low. GRADE ratings were used to guide the strength of interpretation and clinical implications; findings supported by low or very-low certainty evidence were interpreted cautiously [26].

### 2.7. Ethics statement

This study was a systematic review and network meta-analysis based exclusively on aggregate data extracted from previously published studies. No individual participant data were collected, and no new human participants were recruited. Therefore, ethical approval and informed consent were not required.

## 3. Results

### 3.1. Study selection

The study selection process is shown in Fig 1. Electronic database searches identified 4,631 records: 1,498 from the Cochrane Library, 1,423 from Web of Science, 106 from PubMed, 888 from Embase, and 716 from APA PsycInfo. After 1,267 duplicates were removed, 3,364 records were screened; 3,248 were excluded at title/abstract screening and 116 reports underwent full-text assessment. Eighty-eight full-text reports were excluded: ineligible intervention (n = 23), comparator (n = 19), outcome (n = 10), population (n = 17), duplicate study (n = 2), or non-randomized/ineligible design (n = 17). Manual reference-list searching identified 43 records; 39 duplicates or records already captured electronically were removed, four unique records were screened, two were excluded at title/abstract screening, and the remaining two were excluded after full-text review. Thus, the manual branch added no studies. The final review included 28 studies.

**Fig 1:**
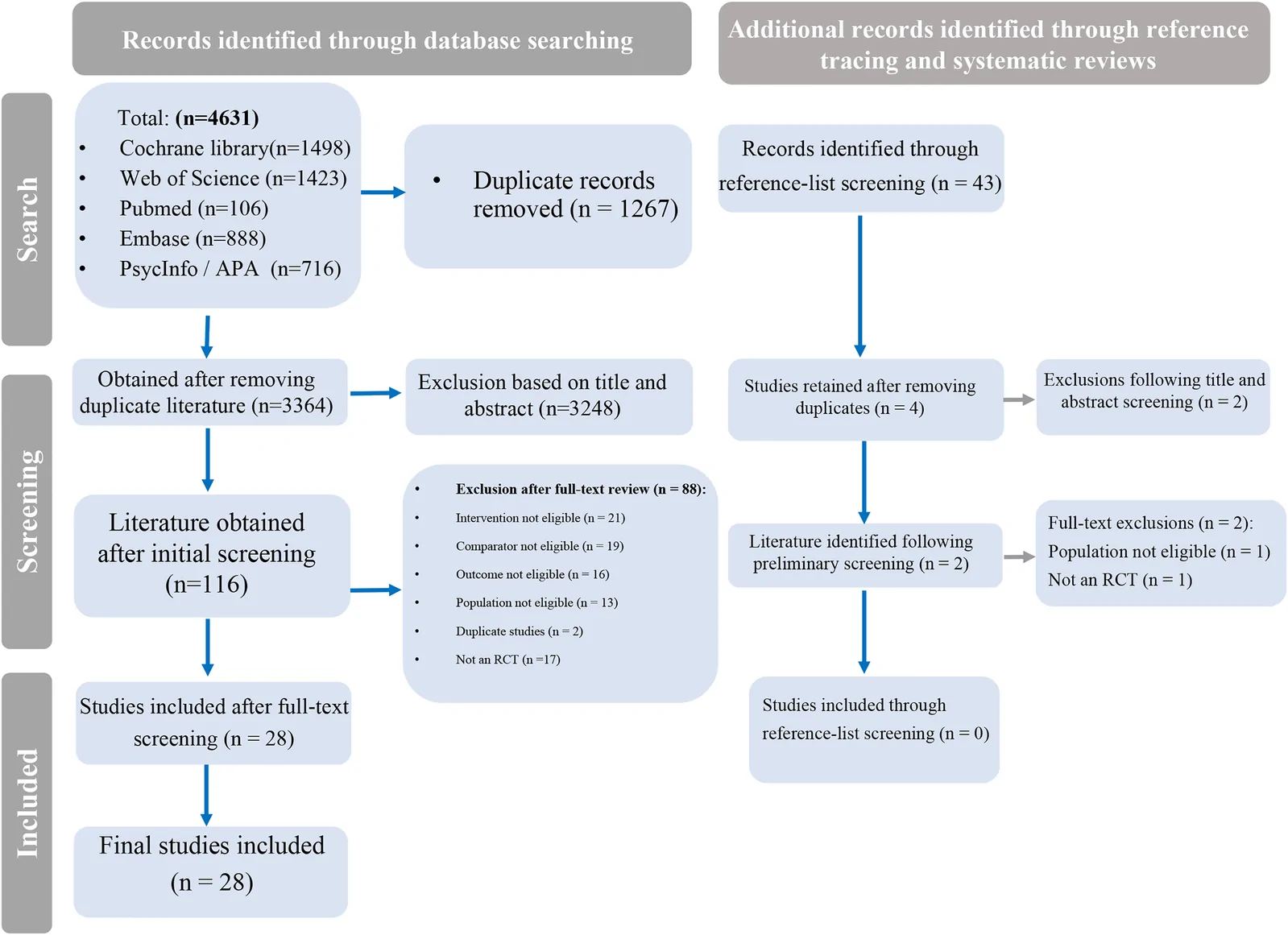
PRISMA flow diagram of literature screening and study selection. Note: Electronic searches identified 4,631 records and yielded 28 included studies. Manual searching identified 43 records; 39 duplicates or records already captured electronically were removed, four unique records were screened, two were excluded at title/abstract screening, and two were excluded after full-text review. The manual branch added no study.

### 3.2. Characteristics of the included studies

The main characteristics of the included studies are presented in S3 File. The 28 studies were published between 2010 and 2025 and included 2,262 participants. These studies were conducted across multiple countries and regions, including Asia, North America, South America, and Europe. All included interventions were chronic exercise interventions. Mean participant age ranged from approximately 60.3 to 86.2 years, and sex distribution varied across studies, with several studies not reporting sex composition. Exercise modalities included aerobic exercise, walking, Tai Chi, yoga, dancing, functional training, multicomponent exercise, resistance exercise, and combined exercise–cognitive programmes. Intervention duration ranged from 6 to 52 weeks, training frequency ranged from 1 to 4 sessions per week, and session duration ranged from 30 to 150 minutes. Assigned MET values ranged from 2.3 to 9.3. Eight studies reported BDNF-related outcomes, nineteen studies reported depression-related outcomes, and one study reported both BDNF and depression-related outcomes. Detailed characteristics of the included studies, including participant demographics, MCI diagnostic criteria, intervention characteristics, comparator conditions, depression measures, BDNF sampling and assay methods, adherence, adverse events, and complex-intervention coding, are provided in S3 File.

### 3.3. Methodological quality of the included studies

The methodological quality assessment of the included studies is presented in S2 Table. Overall, among the 28 randomized controlled trials, 23 were judged to have an overall risk of bias of ‘some concerns’, whereas 5 were rated as ‘high risk’. No study was classified as having an overall ‘low risk’ of bias. Domain-specific assessment showed that the overall performance of D1 (randomization process) was moderate, with 25 studies rated as ‘some concerns’, 2 as ‘low risk’ and 1 as ‘high risk’. In D2 (deviations from intended interventions), 19 studies were rated as ‘some concerns’, 7 as ‘low risk’ and 2 as ‘high risk’. D3 (missing outcome data) emerged as a more prominent source of potential bias, with 15 studies judged as ‘some concerns’, 4 as ‘high risk’ and only 9 as ‘low risk’. By contrast, D4 (measurement of the outcome) showed relatively favourable performance, with 25 studies rated as ‘low risk’ and only 3 as ‘some concerns’. Notably, all 28 studies were judged as having ‘some concerns’ in D5 (selection of the reported result), suggesting that reporting transparency remains a common issue in this field. Taken together, the main methodological limitations of the included studies were not primarily related to outcome measurement, but were more frequently associated with the implementation of randomization, control of deviations from intended interventions, completeness of outcome data and reporting practices.

### 3.4. Network consistency assessment

The results of the node-splitting analyses are presented in S3 Table–S5 Table. Across the three networks, node-splitting analyses did not detect statistically significant differences between direct and indirect evidence for most available comparisons. However, several comparisons were informed by limited direct or indirect evidence, and the analyses may therefore have had limited power to detect disagreement. The absence of statistically significant local inconsistency was not interpreted as confirmation of consistency or transitivity. In the network examining total exercise dose and BDNF, no statistically significant local inconsistency was detected for comparisons between different weekly dose levels and placebo, with all node-splitting P values exceeding 0.05. The directions of the direct, indirect, and model-based estimates were generally similar, suggesting no clear evidence of major disagreement between evidence sources. Nevertheless, the limited number of studies contributing to some dose levels should be considered when interpreting these estimates. In the network assessing total exercise dose and depressive symptoms, no statistically significant local inconsistency was observed for comparisons between each dose level and placebo. However, the comparison between two adjacent moderate-to-high dose levels had a P value closer to the conventional threshold, suggesting potential fluctuation in this part of the evidence base. Therefore, estimates in this dose range should be interpreted with caution. In the modality-specific dose–depression network, node-splitting analyses for the main comparisons involving walking, yoga, Tai Chi, multicomponent exercise, and dancing also showed no statistically significant differences between direct and indirect evidence. Overall, the available analyses did not reveal clear local disagreement, but this finding does not establish consistency or transitivity. Given the sparse evidence for some exercise modalities and dose levels and the incomplete reporting of potential effect modifiers, indirect and modality-specific estimates should be interpreted as exploratory.

### 3.5. Selection of the primary quadratic model and analysis diagnostics

Candidate-model fit was evaluated separately for the two overall outcome networks (S10 Table in S2 File). For BDNF, the Emax model had the lowest deviance information criterion (DIC) value (240.961), followed by the quadratic model (241.166; ΔDIC = 0.205), the linear model (241.873; ΔDIC = 0.912), and the natural cubic spline model (242.512; ΔDIC = 1.551). For overall depressive symptoms, the Emax model again had the lowest DIC (235.703), followed by the linear model (236.717; ΔDIC = 1.014), the quadratic model (236.788; ΔDIC = 1.085), and the natural cubic spline model (238.591; ΔDIC = 2.888).

Considering the prespecified analysis plan, model parsimony, the study objective, and comparative model fit, the quadratic model was retained as the primary descriptive specification. First, it was the originally prespecified main-analysis model and required only two dose–response coefficients, thereby offering a parsimonious representation of the data. Second, it directly represented the single increase-and-decrease pattern relevant to the study objective and allowed estimation of the exercise dose corresponding to the maximum predicted effect [27]. Third, although the Emax model yielded the lowest DIC in both overall outcome networks, its DIC differed only slightly from that of the quadratic model, with ΔDIC values of 0.205 for BDNF and 1.085 for overall depressive symptoms. These small differences did not establish clear statistical superiority of the Emax model. In addition, the linear and Emax models imposed monotonic dose–response patterns and therefore could not represent a possible decline in effect at higher doses. The natural cubic spline model was more flexible but required more parameters and could be less stable or more prone to overfitting in the relatively sparse dose network. Taken together, the model-fit results did not provide sufficient evidence to replace the prespecified quadratic model with an alternative candidate function. The linear, Emax, and natural cubic spline models were therefore used as functional-form sensitivity analyses to assess the stability of the dose-specific predictions from the primary quadratic model rather than as replacements for the primary analysis.

For the primary quadratic descriptive models, the analysis-specific diagnostic results were as follows: for the overall BDNF analysis, DIC = 241.166, mean residual deviance = 21.290, heterogeneity SD = 0.374 (95% credible interval [CrI], 0.078 to 0.865), maximum R-hat = 1.001, and minimum effective sample size (ESS)=1,752; for the overall depressive-symptom analysis, DIC = 236.788, residual deviance = 42.374, heterogeneity SD = 0.185 (95% CrI, 0.022 to 0.343), maximum R-hat = 1.003, and minimum ESS = 684; and for the modality-specific depressive-symptom analysis, DIC = 221.432, residual deviance = 38.847, heterogeneity SD = 0.266 (95% CrI, 0.092 to 0.549), maximum R-hat = 1.004, and minimum ESS = 1,636. All analyses met the prespecified convergence thresholds, indicating satisfactory computational stability of the posterior estimates from the primary quadratic models. Full parameter-level output is provided in S2 Files.

### 3.6. Effects of total exercise dose on BDNF in middle-aged and older adults with MCI

The primary quadratic model suggested a non-linear association between total exercise dose and BDNF in adults with MCI (S6 Table and Fig 2). The posterior mean increased from 110 METs·min/week and reached the largest model-predicted estimate at 440 METs·min/week (mean 0.752, 95% CrI 0.287 to 1.249), followed by a gradual decline at higher doses. At 890 and 1,000 METs·min/week, the 95% CrIs crossed zero, indicating increased uncertainty and limited statistical credibility at the upper dose levels. Thus, 440 METs·min/week is the principal prediction point identified by the selected quadratic model and is interpreted as an exploratory model-based maximum rather than a definitive clinical optimum.

**Fig 2:**
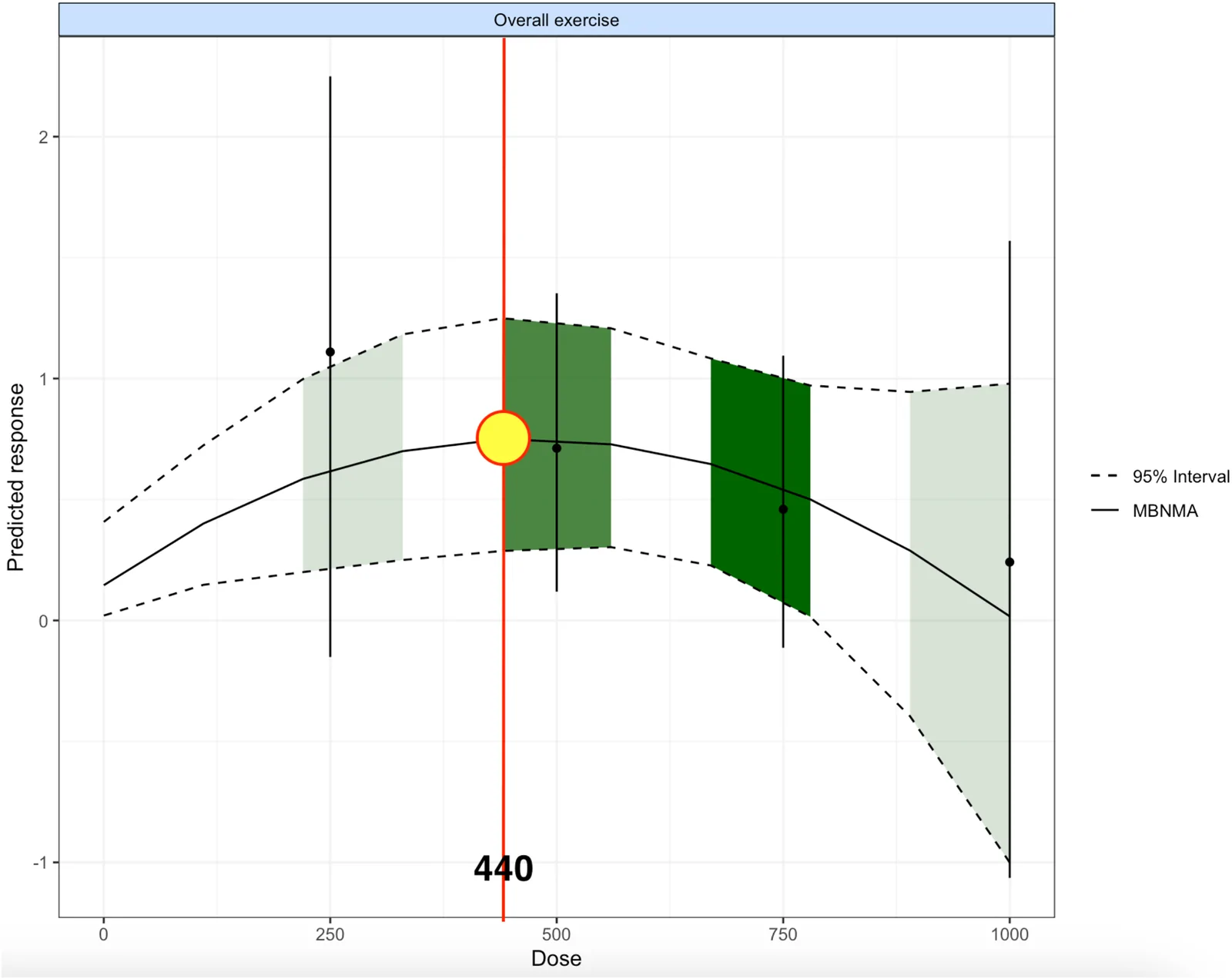
Primary quadratic-model dose–response relationship between overall exercise dose and BDNF in adults with mild cognitive impairment. Note: The figure shows the primary quadratic-model prediction for the effect of overall exercise dose on BDNF. Exercise dose is expressed as METs·min/week, and estimated effects are presented with 95% CrIs.

### 3.7. Dose–response relationship between total exercise dose and depressive symptoms in middle-aged and older adults with MCI

In the primary quadratic model, depressive symptoms also showed an exploratory non-linear dose–response pattern (S7 Table and Fig 3). The largest posterior mean was observed at 670 METs·min/week (mean 0.382, 95% CrI 0.147 to 0.674). At 890 and 1,000 METs·min/week, the 95% CrIs were −0.016 to 0.772 and −0.198 to 0.898, respectively, both crossing zero. Thus, moderate-to-high weekly doses showed comparatively favourable estimates, and 670 METs·min/week is reported as the largest prediction point under the selected quadratic model, rather than as a confirmed therapeutic threshold.

**Fig 3:**
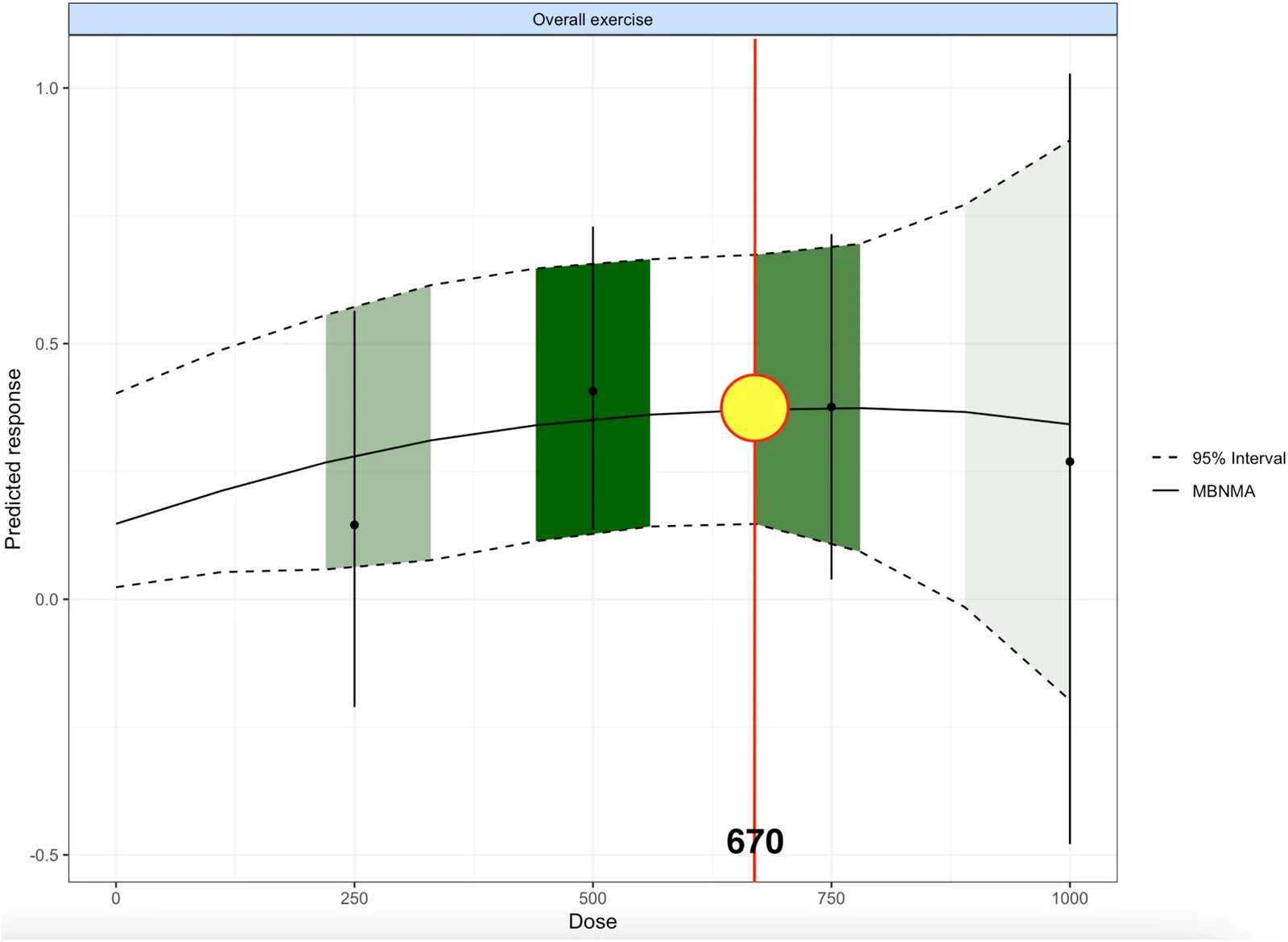
Primary quadratic-model dose–response relationship between overall exercise dose and depressive symptoms in adults with mild cognitive impairment. Note: The figure shows the primary quadratic-model prediction for the effect of overall exercise dose on depressive symptoms. Exercise dose is expressed as METs·min/week, and estimated effects are presented with 95% CrIs.

### 3.8. Effects of exercise modality–dose combinations on depressive symptoms in middle-aged and older adults with MCI

The primary modality-specific quadratic analysis showed heterogeneous non-linear patterns across dancing, multicomponent exercise, Tai Chi, walking, and yoga (S8 Table and Fig 4). Dancing reached comparatively higher estimates at approximately 500–580 METs·min/week, where the 95% CrIs did not cross zero. For multicomponent exercise, the 95% CrIs excluded zero at approximately 670 and 780 METs·min/week, although uncertainty remained at other dose levels, including the point with the largest predicted estimate. For Tai Chi, walking, and yoga, all 95% CrIs crossed zero. These modality-specific predictions therefore suggest favourable dose regions for dancing and multicomponent exercise but remain exploratory because several nodes were sparsely supported. Overall, dancing and multicomponent exercise showed model-predicted improvements at selected dose levels, whereas evidence for Tai Chi, walking, and yoga was more uncertain. These modality-specific findings are secondary to the main quadratic dose–response results and should be considered exploratory rather than definitive.

**Fig 4:**
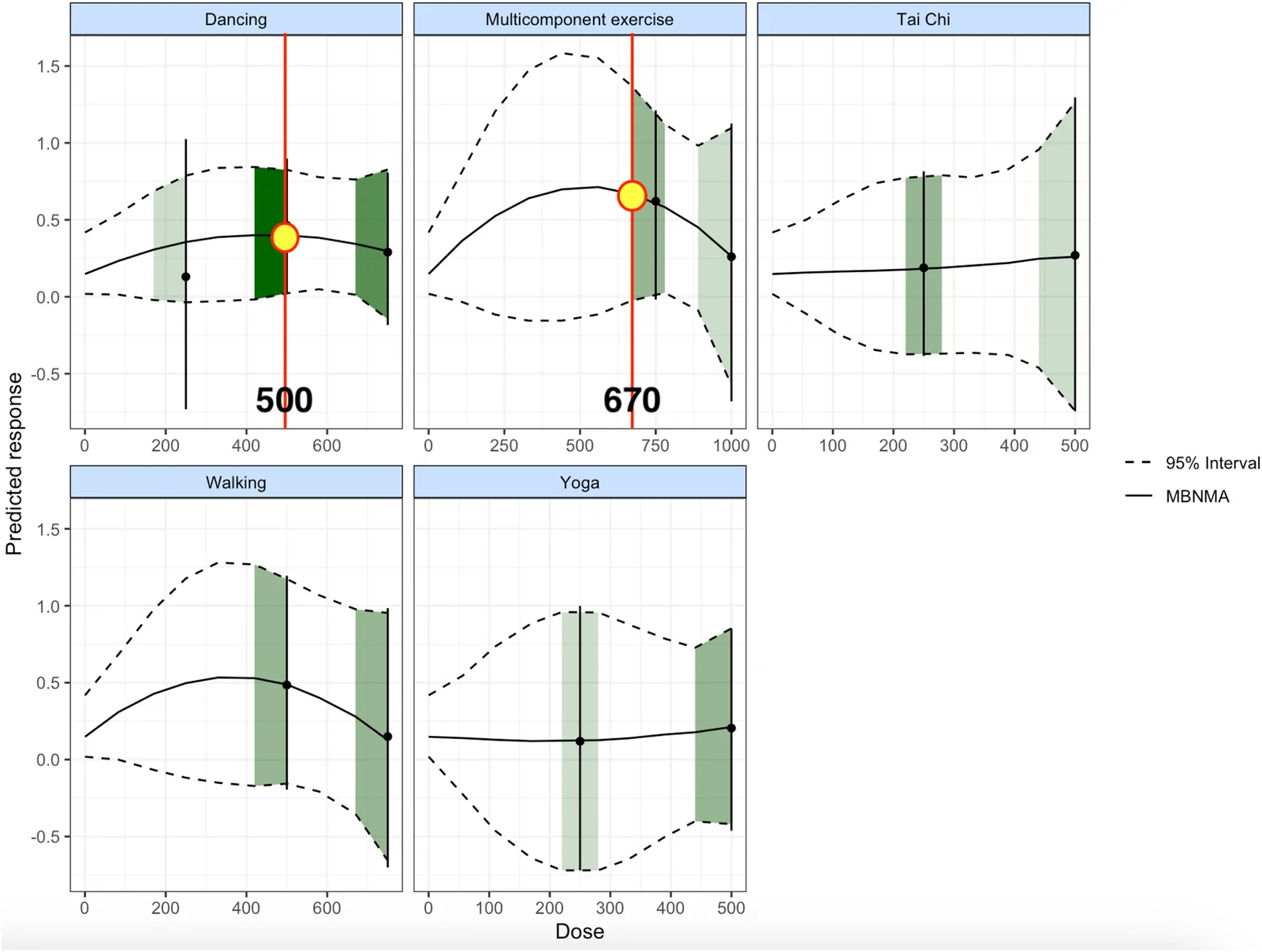
Primary quadratic-model dose–response relationships between exercise modality-specific dose and depressive symptoms in adults with mild cognitive impairment. Note: Primary quadratic-model predictions and 95% CrIs are shown for dancing, multicomponent exercise, Tai Chi, walking, and yoga. Exercise dose is expressed as METs·min/week.

### 3.9. Sensitivity analyses

Functional-form sensitivity was assessed formally by fitting the linear, quadratic, Emax, and natural cubic spline models to each overall network, overlaying the posterior mean dose–response curves, and calculating the posterior distribution of the maximizing dose across the observed range (S11 Fig, S12 Fig, and S10 Table in S2 File). For BDNF, the posterior median maximizing dose was 260 METs·min/week for the natural cubic spline, 470 for the quadratic model, and 1,000 for the linear and Emax models. For depression, the corresponding medians were 700, 910, and 1,000 METs·min/week. The quadratic internal-turning-point intervals were also wide, particularly for depression. These comparisons show that 440 and 670 METs·min/week remain the principal prediction points from the selected quadratic analysis, but their interpretation must be model-specific rather than universal. Study-exclusion sensitivity analyses did not reverse the broad direction of the primary quadratic findings. Thus, sensitivity analysis qualifies the precision of the prediction-point locations without replacing the main quadratic results. Complete diagnostics, priors, code, and data are provided in S2 Files.

### 3.10. Assessment of publication bias

Publication bias and small-study effects were assessed using contour-enhanced funnel plots, Egger’s regression test, the trim-and-fill method, and sunset funnel plots. For the BDNF outcome, the contour-enhanced funnel plot showed a degree of asymmetry (S1-S3 Fig). Egger’s regression test indicated statistically significant funnel plot asymmetry (z = 8.104, P < 0.001), suggesting possible publication bias or small-study effects for this outcome. The trim-and-fill method estimated three potentially missing studies on the left side of the funnel plot (s.e. = 1.980). After imputation of these potentially missing studies, the pooled effect remained statistically significant (estimate = 0.260, s.e. = 0.119, z = 2.180, P = 0.029, 95% CI: 0.026 to 0.493). The sunset funnel plot provides a complementary descriptive display of small-study effects and statistical power (S4 Fig) and was not treated as a formal statistical test. For the depression outcome, the contour-enhanced funnel plot also showed a degree of asymmetry (S5-S9 Fig). Egger’s regression test indicated statistically significant funnel plot asymmetry (z = 3.830, P < 0.001), suggesting possible publication bias or small-study effects. The trim-and-fill analysis estimated that no additional studies were missing on the left side of the funnel plot (s.e. = 1.371). The adjusted pooled effect remained statistically significant (estimate = 0.572, s.e. = 0.049, z = 11.580, P < 0.001, 95% CI: 0.475 to 0.669). The corresponding sunset funnel plot is provided as a descriptive display of small-study effects and statistical power (S10 Fig). Because Egger’s test suggested funnel plot asymmetry, the possibility of publication bias or small-study effects cannot be excluded. Overall, the publication-bias assessments suggested potential asymmetry for both BDNF and depressive symptoms. Although the trim-and-fill analyses did not materially change the direction or statistical significance of the pooled effects, these findings should be interpreted with caution because trim-and-fill adjustment cannot fully rule out selective publication, reporting bias, small-study effects, or heterogeneity-related asymmetry.

### 3.11. Certainty of evidence

The certainty of evidence for the primary outcomes was evaluated using the GRADE approach across five domains: risk of bias, inconsistency, indirectness, imprecision, and publication bias (S9 Table). The certainty ratings were interpreted together with the methodological quality of the included studies, the sparsity of the evidence networks, the width of credible intervals, and the results of publication-bias assessments. For the BDNF outcome, the overall certainty of evidence was rated as low. This rating was mainly attributable to methodological limitations in the included studies, heterogeneity across exercise interventions and dose levels, and evidence suggesting possible publication bias or small-study effects. Although the outcome was directly relevant to the review question, the limited number of studies contributing to some dose levels and the variability in intervention characteristics reduced confidence in the precision and generalisability of the model-predicted estimates.

For depressive symptoms, the overall certainty of evidence was rated as moderate. This outcome was directly relevant to the clinical question, and the direction of the model-predicted effects was generally consistent with a beneficial association between exercise and depressive symptoms. However, certainty was limited by risk of bias in some included studies, heterogeneity in comparator conditions and exercise modalities, sparse evidence for several modality–dose combinations, and possible publication bias or small-study effects. Therefore, the evidence for depressive symptoms should be interpreted as supportive but not definitive. Taken together, the available evidence suggests that exercise may be associated with improvements in depressive symptoms and BDNF in middle-aged and older adults with MCI. However, confidence in these findings differs by outcome and is constrained by methodological limitations, heterogeneity, sparse networks for some comparisons, and potential publication bias. Accordingly, the findings should be regarded as exploratory and hypothesis-generating, and further well-designed randomized controlled trials with transparent intervention reporting, standardized outcome measurement, and complete data reporting are needed to strengthen the evidence base.

## 4. Discussion

### 4.1. Important findings

The main finding of this study is that the primary quadratic model identified exploratory non-linear dose–response patterns for both BDNF and depressive symptoms in middle-aged and older adults with MCI. The largest model-predicted estimates occurred at 440 METs·min/week for BDNF and 670 METs·min/week for depressive symptoms, with greater uncertainty at the upper end of the dose range [28]. These prediction points are the principal findings of the selected quadratic analysis and suggest that larger weekly exercise doses are not necessarily associated with greater improvements [29,30]. Cross-model sensitivity analyses produced different maximizing-dose locations; therefore, these points should be regarded as promising model-based dose regions for future research rather than universally optimal clinical doses [31]. The modality-specific quadratic analysis suggested that depressive-symptom estimates differed across exercise forms. Dancing and multicomponent exercise showed comparatively favourable estimates at selected dose levels, whereas evidence for Tai Chi, walking, and yoga was more uncertain. These findings highlight possible modality-related differences, but the sparse network and wide credible intervals preclude definitive clinical ranking.

Exercise may influence depressive symptoms in adults with MCI through both psychosocial and neurobiological pathways [10]. From a psychosocial perspective, regular exercise may improve self-efficacy, social engagement, perceived competence, and daily functioning, which could contribute to reductions in depressive symptoms [11]. From a neurobiological perspective, exercise may modulate neuroplasticity-related processes, including BDNF signalling, synaptic plasticity, neuronal survival, and activity-dependent adaptation [12]. BDNF has also been implicated in mood regulation and psychiatric disorders, suggesting a plausible link between exercise-induced neurotrophic responses and emotional outcomes [13,14]. However, the present study did not directly test these mechanisms. Therefore, the observed parallel patterns for BDNF and depressive symptoms should be interpreted as hypothesis-generating rather than evidence of a confirmed causal pathway.

From a practical perspective, the findings suggest that future exercise prescriptions for adults with MCI should consider both weekly dose and modality, rather than assuming that higher dose is always preferable. However, because several estimates were imprecise and evidence certainty ranged from low to moderate, the present results should be used to generate hypotheses and guide trial design rather than to prescribe definitive clinical thresholds. Clinicians should individualize exercise programmes according to cognitive status, physical function, comorbidities, safety, tolerance, preference, and adherence.

### 4.2. Possible mechanisms underlying the observed dose-related patterns

The increase-and-decrease pattern predicted by the primary quadratic model raises the possibility that neurobiological and emotional responses to exercise in adults with MCI may be more detectable within a sensitive dose region than at the highest weekly exposure. Previous evidence indicates that exercise can increase peripheral BDNF through neurotrophic support and synaptic plasticity, while the magnitude of response may vary with modality, intensity, duration, age, and baseline condition [28,32]. In individuals with MCI, multicomponent exercise may provide neurobiological, cognitive, and psychosocial stimulation through its combination of aerobic, strength, balance, and task-related elements [33,34]. The favourable estimates for dancing and multicomponent exercise at selected quadratic-model dose nodes are consistent with this possibility, although the aggregate data cannot separate the contribution of each component. The differentiated modality patterns may also reflect rhythmic stimulation, social engagement, intervention content, comparator conditions, and tolerance to exercise. Dancing may combine physical activity with contextual and social engagement, whereas multicomponent programmes integrate several forms of stimulation [35,36]. These mechanisms provide plausible context for the model-predicted patterns but do not establish causal superiority of either modality.

### 4.3. Clinical and practical implications

From a clinical and practical perspective, the quadratic-model prediction points at 440 and 670 METs·min/week provide testable candidate dose regions for future exercise trials targeting BDNF and depressive symptoms, respectively. They should not be converted directly into fixed prescriptions, because functional-form sensitivity and sparse high-dose evidence limit the precision of the predicted maxima. Exercise programmes should be individualized according to cognitive status, physical function, comorbidities, safety, tolerance, preference, feasibility, and adherence.

The favourable estimates across several observed dose levels indicate that the potential benefit of exercise is not confined to one narrowly defined weekly dose. At the same time, the evidence does not support translating the model-specific dose nodes into definitive clinical thresholds. In applied settings, a feasible and sustainable programme tailored to the individual may be more clinically meaningful than targeting a single numerical dose or simply increasing exercise volume.

### 4.4. Limitations and future directions

Several limitations should be acknowledged. First, modality–dose networks were sparse. Dancing and multicomponent exercise showed statistically favourable estimates at selected quadratic-model dose nodes, but many other modality–dose estimates were imprecise and credible intervals widened near the boundaries. The findings therefore support exploratory modality-specific signals rather than definitive modality ranking or fixed recommendations.

Second, comparator conditions were heterogeneous across the included studies. Control conditions included usual care, conventional rehabilitation, stretching or low-intensity activity, physical therapy, activity guidance, and non-exercise cognitive training. These differences may have influenced effect estimates and may also threaten the transitivity assumption required for network meta-analysis. Although transitivity was assessed qualitatively using the available clinical and methodological characteristics, it could not be verified because several potentially important effect modifiers were not consistently reported across studies and some treatment–dose nodes were supported by few studies. Consequently, imbalance in unreported or incompletely reported effect modifiers cannot be excluded, and the indirect and modality-specific estimates may be biased. In addition, non-significant node-splitting results should not be interpreted as proof of consistency because these analyses may have limited power in sparse networks.

Third, the characterization of exercise dose involved uncertainty. Although weekly exercise dose was standardized as METs·min/week where possible, many trials did not provide sufficient detail on exercise intensity, adherence, progression, or the exact time spent in each intervention component. This limitation is particularly relevant for complex interventions, such as multicomponent exercise or programmes combined with cognitive or game-based elements, where the active contribution of each component may be difficult to separate.

Fourth, outcome measurement varied across studies. Depression-related outcomes were assessed using different scales, and baseline depression severity was not consistently reported. For BDNF, sampling source, sampling timing, storage procedures, and assay methods varied across studies. These methodological differences may have contributed to heterogeneity and may have affected the comparability of effect estimates across trials.

Fifth, the analysis was based on aggregate study-level data rather than individual participant data. Therefore, participant-level moderators, such as MCI severity, medication use, comorbidities, baseline physical activity, adherence, and individual response variability, could not be fully examined. In addition, adverse events and intervention adherence were incompletely reported, limiting assessment of feasibility and safety.

Sixth, functional-form uncertainty was material. Cross-model maximizing-dose distributions and overlaid curves showed that internal maxima were not stable across linear, quadratic, Emax, and natural cubic spline models. The quadratic internal-turning-point CrIs were wide, and an internal concave turn was present in only 52.6% of posterior draws for depression. Model-specific turning points should therefore not be interpreted as universally optimal doses. This limitation concerns the precision and generalisability of the optimal-dose location; it does not erase the favourable estimates at several observed dose levels. Exact priors, initialization, software versions, generated JAGS models, analysis data, and parameter-level diagnostics are supplied in S2 Files.

Finally, trial registries and grey-literature sources were not searched; therefore, unpublished or ongoing studies may have been missed. This limitation may have reduced the completeness of the evidence base and increased the risk of publication bias. In addition, funnel-plot asymmetry and Egger’s regression tests suggested possible publication bias or small-study effects for both BDNF and depressive symptoms. Although the trim-and-fill analyses did not materially change the direction of the pooled effects, these analyses cannot fully rule out selective publication, reporting bias, small-study effects, or heterogeneity-related asymmetry. Therefore, the findings should be interpreted cautiously.

Future randomized controlled trials should use standardized diagnostic criteria for MCI, pre-register study protocols, report detailed FITT-VP exercise prescription information [37], and clearly describe comparator conditions. Future studies should also monitor adherence and adverse events, use standardized procedures for measuring BDNF and depressive symptoms, and report sufficient data for dose–response synthesis. Larger, well-powered trials with longer follow-up and harmonized outcome measurement are needed to clarify whether specific exercise modality–dose combinations produce clinically meaningful improvements in BDNF and depressive symptoms in middle-aged and older adults with MCI.

## 5. Conclusion

In conclusion, exercise interventions were associated with increased peripheral BDNF levels and improved depressive symptoms in middle-aged and older adults with mild cognitive impairment, with evidence suggesting a potentially non-linear dose–response relationship. The primary quadratic model indicated that the largest predicted effects on BDNF and depressive symptoms occurred at approximately 440 and 670 METs·min/week, respectively; however, these points should be regarded as candidate dose regions requiring further validation rather than established clinically optimal doses. Dancing and multicomponent exercise showed comparatively favourable effects at selected dose levels, suggesting that exercise benefits may be influenced by both dose and modality. By quantifying the potential effects of exercise on BDNF and depressive symptoms from both dose- and modality-specific perspectives, this study provides new evidence to inform future dose-ranging trials, the optimization of exercise programmes, and the development of more precise exercise intervention strategies for middle-aged and older adults with mild cognitive impairment.

## Supporting information

S1 Data PRISMA Checklist. (DOCX)

S1 Table Search strategies for each database. This table provides the detailed search terms and search procedures used in PubMed, Embase, Web of Science, the Cochrane Library, and APA PsycInfo.
(DOCX)

S2 Table Risk of bias assessment using RoB 2.0. This table presents the risk-of-bias assessment for each included study across the five RoB 2.0 domains and the overall risk-of-bias judgement.
(DOCX)

S3 Table Node-splitting analysis of inconsistency for the overall exercise dose–BDNF network. This table presents the node-splitting results comparing direct and indirect evidence in the overall exercise dose–BDNF network.
(DOCX)

S4 Table Node-splitting analysis of inconsistency for the overall exercise dose–depressive symptoms network. This table presents the node-splitting results comparing direct and indirect evidence in the overall exercise dose–depressive symptoms network.
(DOCX)

S5 Table Node-splitting analysis of inconsistency for the exercise modality-specific dose–depressive symptoms network. This table presents the node-splitting results for the modality-specific dose–depression network.
(DOCX)

S6 Table Quadratic descriptive-model estimates for overall exercise dose and BDNF (effects relative to placebo, E0 = 0). This table presents quadratic descriptive-model posterior estimates relative to placebo (E0 = 0) for BDNF. These values are model-dependent and are not interpreted as optimal-dose estimates.
(DOCX)

S7 Table Quadratic descriptive-model estimates for overall exercise dose and depressive symptoms (effects relative to placebo, E0 = 0). This table presents quadratic descriptive-model posterior estimates relative to placebo (E0 = 0) for depressive symptoms. These values are model-dependent and are not interpreted as optimal-dose estimates.
(DOCX)

S8 Table Modality-specific quadratic descriptive-model estimates for depressive symptoms (effects relative to placebo, E0 = 0). This table presents modality-specific quadratic descriptive-model posterior estimates relative to placebo (E0 = 0). Credible intervals are used to display the substantial uncertainty in sparsely sampled modality–dose combinations.
(DOCX)

S9 Table GRADE assessment of the certainty of evidence. This table summarizes the certainty of evidence for BDNF and depressive symptoms according to the GRADE domains, including risk of bias, inconsistency, indirectness, imprecision, and publication bias.
(DOCX)

S10 Table Candidate-model comparison and analysis-specific Bayesian diagnostics. This table reports the BDNF and overall-depression candidate models separately and provides pD, DIC, within-network ΔDIC, residual deviance, posterior heterogeneity, R-hat, ESS, cross-model maximizing-dose summaries, and quadratic turning-point uncertainty. The same file reports separate diagnostics for the modality-specific depression analysis.
(XLSX)

S1 Fig Posterior mean estimates across overall exercise dose categories for BDNF. The boxplots present the distribution of posterior mean estimates for BDNF across different categories of overall exercise dose.
(DOCX)

S2 Fig Study-specific responses on the link scale across overall exercise dose categories for BDNF. This figure displays the study-specific responses for BDNF on the link scale according to overall exercise dose categories.
(DOCX)

S3 Fig Contour-enhanced funnel plot for publication bias assessment of BDNF outcomes. The contour-enhanced funnel plot displays study comparisons, pseudo 95% confidence limits, and statistical-significance regions for the BDNF outcome.
(DOCX)

S4 Fig Sunset funnel plot for the assessment of small-study effects and statistical power of BDNF outcomes. The sunset funnel plot provides a descriptive display of study precision, small-study effects, and statistical-power regions for the BDNF outcome.
(DOCX)

S5 Fig Posterior mean estimates across overall exercise dose categories for depressive symptoms. The boxplots present the distribution of posterior mean estimates for depressive symptoms across different categories of overall exercise dose.
(DOCX)

S6 Fig Study-specific responses on the link scale across overall exercise dose categories for depressive symptoms. This figure displays the study-specific responses for depressive symptoms on the link scale according to overall exercise dose categories.
(DOCX)

S7 Fig Posterior mean estimates across modality-specific exercise dose categories for depressive symptoms. The boxplots display the distribution of posterior mean estimates for depressive symptoms across modality-specific exercise dose categories.
(DOCX)

S8 Fig Study-specific responses on the link scale across modality-specific exercise dose categories for depressive symptoms. This figure presents study-specific responses for depressive symptoms on the link scale across modality-specific exercise dose categories.
(DOCX)

S9 Fig Contour-enhanced funnel plot for publication bias assessment of depressive symptom outcomes. The contour-enhanced funnel plot displays study comparisons, pseudo 95% confidence limits, and statistical-significance regions for depressive symptom outcomes.
(DOCX)

S10 Fig Sunset funnel plot for the assessment of small-study effects and statistical power of depressive symptom outcomes. The sunset funnel plot provides a descriptive display of study precision, small-study effects, and statistical-power regions for depressive symptom outcomes.
(DOCX)

S11 Fig Functional-form sensitivity analysis for the overall exercise dose–BDNF network. This figure overlays the posterior mean dose–response curves from the linear, quadratic, Emax, and natural cubic spline models for BDNF. It is a sensitivity analysis and does not replace the primary quadratic-model prediction in Fig 2.
(DOCX)

S12 Fig Functional-form sensitivity analysis for the overall exercise dose–depressive symptoms network. This figure overlays the posterior mean dose–response curves from the linear, quadratic, Emax, and natural cubic spline models for depressive symptoms. It is a sensitivity analysis and does not replace the primary quadratic-model prediction in Fig 3.
(DOCX)

S1 File References of studies included in the Bayesian network meta-analysis. This file provides the full reference list of studies included in the Bayesian network meta-analysis.
(DOCX)

S2 File Complete Bayesian analysis data, code, priors, and diagnostics. This file contains the complete arm-level datasets for the three principal networks, full executable R script, exact generated JAGS model code, exact priors, chain initialization, software versions, parameter-level convergence output, analysis-specific DIC/residual-deviance/heterogeneity summaries, cross-model peak-stability results, and S10 Table in S2 File. It is not a code or data excerpt.
(XLSX)

S3 File Detailed characteristics of the included studies and exercise-dose information. This file includes detailed study characteristics, including diagnostic criteria, participant demographics, exercise programmes, assigned MET values, intervention schedules, calculated weekly doses, depression measures, BDNF sampling and assay methods, adherence, adverse events, and source information.
(XLSX)
