# Supplementary material for: Dose- and modality-specific effects of exercise on BDNF and depression in middle-aged and older adults with mild cognitive impairment: A Bayesian network meta-analysis
Source: PLoS One. 2026 Oct 7;21(10):e0358178. doi: 10.1371/journal.pone.0358178 (PMC13645019; doi:10.1371/journal.pone.0358178)
Supplement: Supplementary file 2 [file pone.0358178.s002.docx]

S1 Table. Search strategies for each database.

| **Database** | **Search Terms for Population** | **Search Terms for Intervention** | **Search Terms for Outcomes** |
| --- | --- | --- | --- |
| **Cochrane Library** (limited to trials, N = 1498) | mild cognitive impairment OR MCI: ti, ab, kw | virtual reality OR cycling OR posture OR physical OR locomotion OR stretch OR sprint OR neuromuscular OR balance training OR hydrotherapy OR functional training OR water sports OR yoga OR exergaming OR athletic OR tai chi OR train OR resistance OR motor control OR run OR dance OR activities OR kinesiotherapy OR exercise OR isometric OR endurance OR conditioning OR walk OR treadmill OR mind-body exercise OR strength OR stability OR high intensity interval OR sport OR pilates OR sports combined training OR martial art OR aerobic OR core control OR swim OR activity: ti, ab, kw | brain-derived neurotrophic factor OR BDNF OR depression OR depressive symptoms OR depressive disorder: ti, ab, kw |
| **Web of Science** (limited to Article, N = 1423) | AB=(mild cognitive impairment OR MCI) | AB=(virtual reality OR cycling OR posture OR physical OR locomotion OR stretch OR sprint OR neuromuscular OR balance training OR hydrotherapy OR functional training OR water sports OR yoga OR exergaming OR athletic OR tai chi OR train OR resistance OR motor control OR run OR dance OR activities OR kinesiotherapy OR exercise OR isometric OR endurance OR conditioning OR walk OR treadmill OR mind-body exercise OR strength OR stability OR high intensity interval OR sport OR pilates OR sports combined training OR martial art OR aerobic OR core control OR swim OR activity) | AB=(brain-derived neurotrophic factor OR BDNF OR depression OR depressive symptoms OR depressive disorder) |
| **PubMed** (limited to RCTs OR Clinical Trial, N = 106) | mild cognitive impairment[Title/Abstract] OR MCI[Title/Abstract] | virtual reality[Title/Abstract] OR cycling[Title/Abstract] OR posture[Title/Abstract] OR physical[Title/Abstract] OR locomotion[Title/Abstract] OR stretch[Title/Abstract] OR sprint[Title/Abstract] OR neuromuscular[Title/Abstract] OR balance training[Title/Abstract] OR hydrotherapy[Title/Abstract] OR functional training[Title/Abstract] OR water sports[Title/Abstract] OR yoga[Title/Abstract] OR exergaming[Title/Abstract] OR athletic[Title/Abstract] OR tai chi[Title/Abstract] OR train[Title/Abstract] OR resistance[Title/Abstract] OR motor control[Title/Abstract] OR run[Title/Abstract] OR dance[Title/Abstract] OR activities[Title/Abstract] OR kinesiotherapy[Title/Abstract] OR exercise[Title/Abstract] OR isometric[Title/Abstract] OR endurance[Title/Abstract] OR conditioning[Title/Abstract] OR walk[Title/Abstract] OR treadmill[Title/Abstract] OR mind-body exercise[Title/Abstract] OR strength[Title/Abstract] OR stability[Title/Abstract] OR high intensity interval[Title/Abstract] OR sport[Title/Abstract] OR pilates[Title/Abstract] OR sports combined training[Title/Abstract] OR martial art[Title/Abstract] OR aerobic[Title/Abstract] OR core control[Title/Abstract] OR swim[Title/Abstract] OR activity[Title/Abstract] | brain-derived neurotrophic factor[Title/Abstract] OR BDNF[Title/Abstract] OR depression[Title/Abstract] OR depressive symptoms [Title/Abstract] OR depressive disorder[Title/Abstract] |
| **Embase** (limited to controlled study, N = 888) | 'mild cognitive impairment':ab,ti OR mci:ab,ti | 'virtual reality':ab,ti OR cycling:ab,ti OR posture:ab,ti OR physical:ab,ti OR locomotion:ab,ti OR stretch:ab,ti OR sprint:ab,ti OR neuromuscular:ab,ti OR 'balance training':ab,ti OR hydrotherapy:ab,ti OR 'functional training':ab,ti OR 'water sports':ab,ti OR yoga:ab,ti OR exergaming:ab,ti OR athletic:ab,ti OR 'tai chi':ab,ti OR train:ab,ti OR resistance:ab,ti OR 'motor control':ab,ti OR run:ab,ti OR dance:ab,ti OR activities:ab,ti OR kinesiotherapy:ab,ti OR exercise:ab,ti OR isometric:ab,ti OR endurance:ab,ti OR conditioning:ab,ti OR walk:ab,ti OR treadmill:ab,ti OR 'mind-body exercise':ab,ti OR strength:ab,ti OR stability:ab,ti OR 'high intensity interval':ab,ti OR sport:ab,ti OR pilates:ab,ti OR 'sports combined training':ab,ti OR 'martial art':ab,ti OR aerobic:ab,ti OR 'core control':ab,ti OR swim:ab,ti OR activity:ab,ti | 'brain-derived neurotrophic factor':ti,ab,kw OR bdnf:ti,ab,kw) AND (depression:ti,ab,kw OR 'depressive symptoms':ti,ab,kw OR 'depressive disorder':ti,ab,kw |
| **PsycInfo / APA** (N = 716) | Abstract: mild cognitive impairment OR MCI | Abstract: virtual reality OR cycling OR posture OR physical OR locomotion OR stretch OR sprint OR neuromuscular OR balance training OR hydrotherapy OR functional training OR water sports OR yoga OR exergaming OR athletic OR tai chi OR train OR resistance OR motor control OR run OR dance OR activities OR kinesiotherapy OR exercise OR isometric OR endurance OR conditioning OR walk OR treadmill OR mind-body exercise OR strength OR stability OR high intensity interval OR sport OR pilates OR sports combined training OR martial art OR aerobic OR core control OR swim OR activity | Abstract: brain-derived neurotrophic factor OR BDNF OR depression OR depressive symptoms OR depressive disorder |
