# Supplementary material for: Dose- and modality-specific effects of exercise on BDNF and depression in middle-aged and older adults with mild cognitive impairment: A Bayesian network meta-analysis
Source: PLoS One. 2026 Oct 7;21(10):e0358178. doi: 10.1371/journal.pone.0358178 (PMC13645019; doi:10.1371/journal.pone.0358178)
Supplement: Supplementary file 3 [file pone.0358178.s003.docx]

S2 Table. Risk of bias assessment using RoB 2.0

| **Study** | **D1** | **D2** | **D3** | **D4** | **D5** | **Overall** |
| --- | --- | --- | --- | --- | --- | --- |
| Baker (2010, USA) | Some concerns | Some concerns | Some concerns | Low | Some concerns | Some concerns |
| Allard (2017, USA) | Some concerns | Some concerns | Some concerns | Low | Some concerns | Some concerns |
| Kohanpour (2017, Iran) | Some concerns | Some concerns | Some concerns | Low | Some concerns | Some concerns |
| Damirchi (2018, Iran) | Some concerns | Some concerns | Some concerns | Some concerns | Some concerns | Some concerns |
| Yang (2016, USA) | Some concerns | Some concerns | High | Low | Some concerns | High |
| Eyre (2017, USA) | Some concerns | Some concerns | Some concerns | Low | Some concerns | Some concerns |
| Lazarou (2017, Greece) | Some concerns | Some concerns | Some concerns | Low | Some concerns | Some concerns |
| Sungkarat (2018, Thailand) | Some concerns | Low | Low | Low | Some concerns | Some concerns |
| Zhu (2018, China) | Some concerns | Low | Low | Low | Some concerns | Some concerns |
| Langoni (2019, Brazil) | Some concerns | Some concerns | Low | Low | Some concerns | Some concerns |
| Tsai (2019, Taiwan) | Some concerns | High | High | Some concerns | Some concerns | High |
| Song (2019, China) | Some concerns | Low | Low | Low | Some concerns | Some concerns |
| Lam (2012, Hong Kong, China) | Some concerns | Some concerns | High | Low | Some concerns | High |
| Lam (2015, Hong Kong, China) | Some concerns | Some concerns | Some concerns | Low | Some concerns | Some concerns |
| Wang (2020, China) structured limbs-exercise | Some concerns | Low | Low | Low | Some concerns | Some concerns |
| Bisbe (2020, Spain) | Low | Some concerns | Low | Low | Some concerns | Some concerns |
| Wang (2020, China) square dancing | Low | Some concerns | Low | Low | Some concerns | Some concerns |
| Ho (2020, Hong Kong, China) | Some concerns | Some concerns | Some concerns | Some concerns | Some concerns | Some concerns |
| Chang (2021, China) | High | Some concerns | Some concerns | Low | Some concerns | High |
| Tremont (2022, USA) | Some concerns | Some concerns | Some concerns | Low | Some concerns | Some concerns |
| Yu (2022, Hong Kong, China) | Some concerns | Low | Low | Low | Some concerns | Some concerns |
| Zhang (2023, China) | Some concerns | Some concerns | Some concerns | Low | Some concerns | Some concerns |
| Khanthong (2024, Thailand) | Some concerns | Low | Low | Low | Some concerns | Some concerns |
| Sánchez-Alcalá (2024, Spain) | Some concerns | High | High | Low | Some concerns | High |
| Zhang (2024, China) | Some concerns | Some concerns | Some concerns | Low | Some concerns | Some concerns |
| Buele (2024, Ecuador/Spain collaboration) | Some concerns | Low | Some concerns | Low | Some concerns | Some concerns |
| Lin (2025, China) | Some concerns | Some concerns | Some concerns | Low | Some concerns | Some concerns |
| Resende-Silva (2025, Brazil) | Some concerns | Some concerns | Some concerns | Low | Some concerns | Some concerns |

**Notes:** Risk of bias was assessed using the RoB 2.0 tool; D1 = bias arising from the randomization process; D2 = bias due to deviations from intended interventions; D3 = bias due to missing outcome data; D4 = bias in measurement of the outcome; D5 = bias in selection of the reported result; Overall = overall risk of bias.
