# Supplementary material for: Dose- and modality-specific effects of exercise on BDNF and depression in middle-aged and older adults with mild cognitive impairment: A Bayesian network meta-analysis
Source: PLoS One. 2026 Oct 7;21(10):e0358178. doi: 10.1371/journal.pone.0358178 (PMC13645019; doi:10.1371/journal.pone.0358178)
Supplement: Supplementary file 4 [file pone.0358178.s004.docx]

S3 Table. Node-splitting analysis of inconsistency for the overall exercise dose–BDNF network

| Comparison | p-value | Median | 2.50% | 97.50% |
| --- | --- | --- | --- | --- |
| Overall exercise_1000 vs Placebo_0 | 0.493 |  |  |  |
| -> direct |  | 0.082 | -1.372 | 1.426 |
| -> indirect |  | 0.683 | -0.017 | 1.356 |
| -> MBNMA |  | 0.569 | -0.032 | 1.152 |
|  |  |  |  |  |
| Overall exercise_750 vs Placebo_0 | 0.648 |  |  |  |
| -> direct |  | 0.294 | -0.331 | 0.935 |
| -> indirect |  | 0.568 | -0.078 | 1.25 |
| -> MBNMA |  | 0.427 | -0.024 | 0.864 |
|  |  |  |  |  |
| Overall exercise_500 vs Placebo_0 | 0.382 |  |  |  |
| -> direct |  | 0.564 | 0.005 | 1.164 |
| -> indirect |  | 0.187 | -0.167 | 0.523 |
| -> MBNMA |  | 0.285 | -0.016 | 0.576 |
|  |  |  |  |  |
| Overall exercise_250 vs Placebo_0 | 0.109 |  |  |  |
| -> direct |  | 0.914 | -0.287 | 2.115 |
| -> indirect |  | 0.131 | -0.017 | 0.276 |
| -> MBNMA |  | 0.142 | -0.008 | 0.288 |
