# Supplementary material for: Dose- and modality-specific effects of exercise on BDNF and depression in middle-aged and older adults with mild cognitive impairment: A Bayesian network meta-analysis
Source: PLoS One. 2026 Oct 7;21(10):e0358178. doi: 10.1371/journal.pone.0358178 (PMC13645019; doi:10.1371/journal.pone.0358178)
Supplement: Supplementary file 5 [file pone.0358178.s005.docx]

S4 Table. Node-splitting analysis of inconsistency for the overall exercise dose–depressive symptoms network

| Comparison | p-value | Median | 2.50% | 97.50% |
| --- | --- | --- | --- | --- |
| Overall exercise_750 vs Overall exercise_500 | 0.077 |  |  |  |
| -> direct |  | -0.557 | -1.697 | 0.602 |
| -> indirect |  | 0.083 | 0.018 | 0.143 |
| -> MBNMA |  | 0.082 | 0.01 | 0.145 |
|  |  |  |  |  |
| Overall exercise_1000 vs Placebo_0 | 0.482 |  |  |  |
| -> direct |  | 0.103 | -0.622 | 0.815 |
| -> indirect |  | 0.358 | 0.063 | 0.623 |
| -> MBNMA |  | 0.329 | 0.041 | 0.578 |
|  |  |  |  |  |
| Overall exercise_250 vs Placebo_0 | 0.316 |  |  |  |
| -> direct |  | -0.012 | -0.332 | 0.314 |
| -> indirect |  | 0.084 | 0.015 | 0.147 |
| -> MBNMA |  | 0.082 | 0.01 | 0.145 |
