# Supplementary material for: Dose- and modality-specific effects of exercise on BDNF and depression in middle-aged and older adults with mild cognitive impairment: A Bayesian network meta-analysis
Source: PLoS One. 2026 Oct 7;21(10):e0358178. doi: 10.1371/journal.pone.0358178 (PMC13645019; doi:10.1371/journal.pone.0358178)
Supplement: Supplementary file 6 [file pone.0358178.s006.docx]

S5 Table. Node-splitting analysis of inconsistency for the exercise modality-specific dose–depressive symptoms network

| Comparison | p-value | Median | 2.50% | 97.50% |
| --- | --- | --- | --- | --- |
| Walking_750 vs Walking_500 | 0.228 |  |  |  |
| -> direct |  | -0.528 | -1.738 | 0.602 |
| -> indirect |  | 0.084 | -0.142 | 0.299 |
| -> MBNMA |  | 0.065 | -0.157 | 0.269 |
|  |  |  |  |  |
| Yoga_500 vs Placebo_0 | 0.531 |  |  |  |
| -> direct |  | 0.051 | -0.52 | 0.602 |
| -> indirect |  | -0.124 | -1.704 | 1.467 |
| -> MBNMA |  | 0.026 | -0.505 | 0.524 |
|  |  |  |  |  |
| Yoga_250 vs Placebo_0 | 0.553 |  |  |  |
| -> direct |  | -0.046 | -0.834 | 0.698 |
| -> indirect |  | 0.024 | -0.258 | 0.306 |
| -> MBNMA |  | 0.013 | -0.252 | 0.262 |
|  |  |  |  |  |
| Tai Chi_500 vs Placebo_0 | 0.962 |  |  |  |
| -> direct |  | 0.084 | -0.877 | 1.038 |
| -> indirect |  | 0.049 | -0.904 | 1.096 |
| -> MBNMA |  | 0.065 | -0.582 | 0.713 |
|  |  |  |  |  |
| Tai Chi_250 vs Placebo_0 | 0.94 |  |  |  |
| -> direct |  | 0.02 | -0.493 | 0.539 |
| -> indirect |  | 0.051 | -0.396 | 0.506 |
| -> MBNMA |  | 0.032 | -0.291 | 0.356 |
|  |  |  |  |  |
| Multicomponent exercise_1000 vs Placebo_0 | 0.44 |  |  |  |
| -> direct |  | 0.097 | -0.653 | 0.857 |
| -> indirect |  | 0.627 | -0.028 | 1.235 |
| -> MBNMA |  | 0.412 | -0.074 | 0.871 |
|  |  |  |  |  |
| Multicomponent exercise_750 vs Placebo_0 | 0.453 |  |  |  |
| -> direct |  | 0.461 | -0.037 | 0.898 |
| -> indirect |  | 0.083 | -0.489 | 0.635 |
| -> MBNMA |  | 0.309 | -0.055 | 0.653 |
|  |  |  |  |  |
| Dancing_750 vs Placebo_0 | 0.45 |  |  |  |
| -> direct |  | 0.118 | -0.269 | 0.506 |
| -> indirect |  | 0.43 | -0.051 | 0.895 |
| -> MBNMA |  | 0.243 | -0.075 | 0.537 |
|  |  |  |  |  |
| Dancing_500 vs Placebo_0 | 0.416 |  |  |  |
| -> direct |  | 0.303 | -0.037 | 0.619 |
| -> indirect |  | 0.072 | -0.195 | 0.329 |
| -> MBNMA |  | 0.162 | -0.05 | 0.358 |
|  |  |  |  |  |
| Dancing_250 vs Placebo_0 | 0.266 |  |  |  |
| -> direct |  | -0.026 | -0.778 | 0.694 |
| -> indirect |  | 0.081 | -0.03 | 0.185 |
| -> MBNMA |  | 0.081 | -0.025 | 0.179 |
