# Supplementary material for: Dose- and modality-specific effects of exercise on BDNF and depression in middle-aged and older adults with mild cognitive impairment: A Bayesian network meta-analysis
Source: PLoS One. 2026 Oct 7;21(10):e0358178. doi: 10.1371/journal.pone.0358178 (PMC13645019; doi:10.1371/journal.pone.0358178)
Supplement: Supplementary file 7 [file pone.0358178.s007.docx]

S6 Table. Model-predicted estimates for the effects of overall exercise dose on BDNF

| agent | dose | mean | sd | 2.50% | 25% | 50% | 75% | 97.50% |
| --- | --- | --- | --- | --- | --- | --- | --- | --- |
| Overall exercise | 0 | 0.1655695 | 0.1030466 | 0.02018601 | 0.086329 | 0.14556426 | 0.2270231 | 0.4073151 |
| Overall exercise | 110 | 0.40866889 | 0.1494366 | 0.14724979 | 0.30315773 | 0.40054098 | 0.4995039 | 0.723938 |
| Overall exercise | 220 | 0.58748057 | 0.2062609 | 0.1998569 | 0.4472463 | 0.5852962 | 0.7145619 | 0.9980459 |
| Overall exercise | 330 | 0.70200453 | 0.2394362 | 0.25000777 | 0.54050994 | 0.69994822 | 0.8512882 | 1.1827959 |
| Overall exercise | 440 | 0.75224077 | 0.2460425 | 0.28742291 | 0.58660139 | 0.75009536 | 0.9075467 | 1.2493282 |
| Overall exercise | 560 | 0.73372407 | 0.23009 | 0.30301885 | 0.58536044 | 0.72813972 | 0.8804806 | 1.207557 |
| Overall exercise | 670 | 0.64954054 | 0.2151915 | 0.2274051 | 0.51340369 | 0.64662566 | 0.7887775 | 1.0833433 |
| Overall exercise | 780 | 0.50106929 | 0.2412882 | 0.0150759 | 0.35130489 | 0.50024969 | 0.6538842 | 0.9711913 |
| Overall exercise | 890 | 0.28831032 | 0.3381198 | -0.39615685 | 0.07172924 | 0.28870161 | 0.5097249 | 0.9448203 |
| Overall exercise | 1000 | 0.01126364 | 0.4980373 | -1.00071384 | -0.31298701 | 0.01716162 | 0.3307659 | 0.9788279 |
