# Supplementary material for: Dose- and modality-specific effects of exercise on BDNF and depression in middle-aged and older adults with mild cognitive impairment: A Bayesian network meta-analysis
Source: PLoS One. 2026 Oct 7;21(10):e0358178. doi: 10.1371/journal.pone.0358178 (PMC13645019; doi:10.1371/journal.pone.0358178)
Supplement: Supplementary file 8 [file pone.0358178.s008.docx]

S7 Table. Model-predicted estimates for the effects of overall exercise dose on depressive symptoms

| agent | dose | mean | sd | 2.50% | 25% | 50% | 75% | 97.50% |
| --- | --- | --- | --- | --- | --- | --- | --- | --- |
| Overall exercise | 0 | 0.1646527 | 0.09966969 | 0.0235383 | 0.08826813 | 0.1475623 | 0.2229269 | 0.4024805 |
| Overall exercise | 110 | 0.2275149 | 0.10996657 | 0.05303442 | 0.1463829 | 0.2118064 | 0.2939422 | 0.4873422 |
| Overall exercise | 220 | 0.2796946 | 0.12610232 | 0.05833884 | 0.1907301 | 0.2676672 | 0.3570195 | 0.5559274 |
| Overall exercise | 330 | 0.3211917 | 0.13608583 | 0.07644434 | 0.22707962 | 0.3107986 | 0.4050436 | 0.6143739 |
| Overall exercise | 440 | 0.3520062 | 0.13734281 | 0.11348431 | 0.25781589 | 0.3403047 | 0.4350494 | 0.6470308 |
| Overall exercise | 560 | 0.3734385 | 0.1322417 | 0.14227896 | 0.28388034 | 0.3610258 | 0.4544171 | 0.664988 |
| Overall exercise | 670 | 0.3819167 | 0.13166109 | 0.14732971 | 0.29103142 | 0.3705554 | 0.4584507 | 0.6736974 |
| Overall exercise | 780 | 0.3797123 | 0.15062793 | 0.09322703 | 0.28096228 | 0.373846 | 0.4739036 | 0.6952165 |
| Overall exercise | 890 | 0.3668253 | 0.19916685 | -0.01569501 | 0.23675827 | 0.3663481 | 0.4964523 | 0.7718085 |
| Overall exercise | 1000 | 0.3432557 | 0.27594868 | -0.19750158 | 0.16327008 | 0.3422441 | 0.5205165 | 0.8978298 |
