# Supplementary material for: Dose- and modality-specific effects of exercise on BDNF and depression in middle-aged and older adults with mild cognitive impairment: A Bayesian network meta-analysis
Source: PLoS One. 2026 Oct 7;21(10):e0358178. doi: 10.1371/journal.pone.0358178 (PMC13645019; doi:10.1371/journal.pone.0358178)
Supplement: Supplementary file 9 [file pone.0358178.s009.docx]

S8 Table. Model-predicted estimates for the effects of modality-specific exercise dose on depressive symptoms

| agent | dose | mean | sd | 2.50% | 25% | 50% | 75% | 97.50% |
| --- | --- | --- | --- | --- | --- | --- | --- | --- |
| Dancing | 0 | 0.1666276 | 0.1015494 | 0.022972377 | 0.087736141 | 0.1503416 | 0.2283967 | 0.4123398 |
| Dancing | 83 | 0.2473788 | 0.1314297 | 0.01488664 | 0.15682644 | 0.2400401 | 0.3265176 | 0.5294035 |
| Dancing | 170 | 0.3146646 | 0.1775394 | -0.02619207 | 0.198459672 | 0.3133884 | 0.4251622 | 0.6819595 |
| Dancing | 250 | 0.3608577 | 0.2074352 | -0.048174689 | 0.225879592 | 0.3658844 | 0.4914194 | 0.7773487 |
| Dancing | 330 | 0.3920291 | 0.2206667 | -0.043297082 | 0.248248179 | 0.397836 | 0.5325259 | 0.8385685 |
| Dancing | 420 | 0.4091413 | 0.2161432 | -0.029996122 | 0.271497053 | 0.4137497 | 0.5455279 | 0.8458983 |
| Dancing | 500 | 0.4083916 | 0.1992334 | 0.017683999 | 0.279020065 | 0.4087464 | 0.5348782 | 0.8158758 |
| Dancing | 580 | 0.3926202 | 0.1813968 | 0.041407006 | 0.272036613 | 0.389688 | 0.5070375 | 0.7631195 |
| Dancing | 670 | 0.3569218 | 0.1888216 | -0.006225804 | 0.235981472 | 0.3545882 | 0.4748695 | 0.7479066 |
| Dancing | 750 | 0.3092293 | 0.2442875 | -0.163629454 | 0.154646539 | 0.3090269 | 0.4568283 | 0.7929533 |
| Multicomponent exercise | 0 | 0.1666276 | 0.1015494 | 0.022972377 | 0.087736141 | 0.1503416 | 0.2283967 | 0.4123398 |
| Multicomponent exercise | 110 | 0.3761878 | 0.2027695 | -0.015254348 | 0.241013549 | 0.3691283 | 0.5129425 | 0.7703319 |
| Multicomponent exercise | 220 | 0.5366154 | 0.3180603 | -0.095733178 | 0.328351682 | 0.5321639 | 0.7516295 | 1.1552208 |
| Multicomponent exercise | 330 | 0.6479106 | 0.3903778 | -0.135323518 | 0.391750065 | 0.6475249 | 0.908592 | 1.396703 |
| Multicomponent exercise | 440 | 0.7100733 | 0.415633 | -0.121446937 | 0.441727211 | 0.7085996 | 0.9864974 | 1.5068179 |
| Multicomponent exercise | 560 | 0.7218517 | 0.3912055 | -0.057303434 | 0.469644944 | 0.7215055 | 0.9818321 | 1.4664617 |
| Multicomponent exercise | 670 | 0.6812828 | 0.3286815 | 0.020491388 | 0.469763997 | 0.685944 | 0.9047064 | 1.2963064 |
| Multicomponent exercise | 780 | 0.5915814 | 0.2551158 | 0.078274283 | 0.428209994 | 0.590453 | 0.7583134 | 1.0707325 |
| Multicomponent exercise | 890 | 0.4527475 | 0.2599523 | -0.052289673 | 0.283417137 | 0.4523722 | 0.621209 | 0.9451359 |
| Multicomponent exercise | 1000 | 0.2647812 | 0.4181827 | -0.565847566 | 0.001419638 | 0.2711996 | 0.5274865 | 1.0593148 |
| Tai Chi | 0 | 0.1666276 | 0.1015494 | 0.022972377 | 0.087736141 | 0.1503416 | 0.2283967 | 0.4123398 |
| Tai Chi | 56 | 0.1672306 | 0.152769 | -0.118630458 | 0.064785769 | 0.1607881 | 0.2569873 | 0.490987 |
| Tai Chi | 110 | 0.1702146 | 0.2174393 | -0.25668829 | 0.032175741 | 0.1676071 | 0.297169 | 0.6185915 |
| Tai Chi | 170 | 0.1762968 | 0.2644894 | -0.348834624 | 0.005617686 | 0.1747984 | 0.3358421 | 0.7199995 |
| Tai Chi | 220 | 0.1835898 | 0.2811775 | -0.373713686 | 0.003723522 | 0.1779275 | 0.3545092 | 0.7564978 |
| Tai Chi | 280 | 0.1950109 | 0.2798655 | -0.348901099 | 0.015639877 | 0.1891931 | 0.3676346 | 0.7557713 |
| Tai Chi | 330 | 0.2067531 | 0.2729048 | -0.3097222 | 0.023724766 | 0.2026692 | 0.3789536 | 0.758028 |
| Tai Chi | 390 | 0.2235133 | 0.28711 | -0.303502312 | 0.021202527 | 0.2201175 | 0.4115392 | 0.7944903 |
| Tai Chi | 440 | 0.2397046 | 0.3447644 | -0.409555251 | 0.005134382 | 0.2350686 | 0.4661496 | 0.9453142 |
| Tai Chi | 500 | 0.2618037 | 0.480794 | -0.696233568 | -0.057485541 | 0.2560412 | 0.5731741 | 1.217258 |
| Walking | 0 | 0.1666276 | 0.1015494 | 0.022972377 | 0.087736141 | 0.1503416 | 0.2283967 | 0.4123398 |
| Walking | 83 | 0.308642 | 0.1729594 | -0.021540045 | 0.193491505 | 0.3002085 | 0.4211663 | 0.6603006 |
| Walking | 170 | 0.4190876 | 0.2672431 | -0.119485375 | 0.244988031 | 0.4124708 | 0.5914838 | 0.9486744 |
| Walking | 250 | 0.4859478 | 0.3269897 | -0.167685244 | 0.274207805 | 0.4845442 | 0.6945307 | 1.1329355 |
| Walking | 330 | 0.5195635 | 0.3573572 | -0.202502456 | 0.285843047 | 0.518092 | 0.7475066 | 1.2255317 |
| Walking | 420 | 0.5176437 | 0.3591297 | -0.185850621 | 0.286968419 | 0.5147649 | 0.7438 | 1.2264001 |
| Walking | 500 | 0.4806149 | 0.3395999 | -0.167841445 | 0.263603982 | 0.4781293 | 0.6939345 | 1.1478032 |
| Walking | 580 | 0.4103416 | 0.3173863 | -0.21274693 | 0.209217257 | 0.4053382 | 0.6147 | 1.0235675 |
| Walking | 670 | 0.2915467 | 0.3301142 | -0.348043163 | 0.086291252 | 0.2876548 | 0.5111669 | 0.9119531 |
| Walking | 750 | 0.150629 | 0.4123553 | -0.663839141 | -0.118642092 | 0.1410035 | 0.4252699 | 0.9467427 |
| Yoga | 0 | 0.1666276 | 0.1015494 | 0.022972377 | 0.087736141 | 0.1503416 | 0.2283967 | 0.4123398 |
| Yoga | 56 | 0.1432257 | 0.199305 | -0.248731782 | 0.017040898 | 0.1397901 | 0.2717546 | 0.5437739 |
| Yoga | 110 | 0.1274834 | 0.3120806 | -0.497539375 | -0.070029413 | 0.1274553 | 0.3262927 | 0.7388581 |
| Yoga | 170 | 0.1178499 | 0.3975681 | -0.684291736 | -0.127745969 | 0.1201172 | 0.374497 | 0.9094233 |
| Yoga | 220 | 0.1161403 | 0.4329709 | -0.749555983 | -0.14821281 | 0.124015 | 0.393719 | 0.9763465 |
| Yoga | 280 | 0.121671 | 0.4332112 | -0.761302222 | -0.144595173 | 0.1286659 | 0.3973126 | 0.9755361 |
| Yoga | 330 | 0.1325983 | 0.4014752 | -0.707298898 | -0.111322598 | 0.1388867 | 0.3906715 | 0.9195694 |
| Yoga | 390 | 0.1532931 | 0.3371027 | -0.537426633 | -0.052520796 | 0.1607702 | 0.3662066 | 0.7940185 |
| Yoga | 440 | 0.1768573 | 0.2894723 | -0.421640876 | -0.002951694 | 0.1803526 | 0.3624098 | 0.7411004 |
| Yoga | 500 | 0.2127164 | 0.3191944 | -0.429867419 | 0.007927253 | 0.213781 | 0.4073451 | 0.8429531 |
