# Supplementary material for: Dose- and modality-specific effects of exercise on BDNF and depression in middle-aged and older adults with mild cognitive impairment: A Bayesian network meta-analysis
Source: PLoS One. 2026 Oct 7;21(10):e0358178. doi: 10.1371/journal.pone.0358178 (PMC13645019; doi:10.1371/journal.pone.0358178)
Supplement: Supplementary file 10 [file pone.0358178.s010.docx]

S9 Table. GRADE assessment of the certainty of evidence

| Outcome | Risk of bias | Inconsistency | Indirectness | Imprecision | Publication bias | Overall certainty |
| --- | --- | --- | --- | --- | --- | --- |
| BDNF | Serious | Serious | Not serious | Not serious | Undetected | Low |
| Depressive symptoms | Serious | Not serious | Not serious | Not serious | Undetected | Moderate |

**Notes:** GRADE certainty was assessed according to risk of bias, inconsistency, indirectness, imprecision, and publication bias. The certainty of evidence was rated as low for BDNF, mainly because of methodological limitations and heterogeneity across interventions, and moderate for depressive symptoms, as the direction of effect was generally consistent and the outcome was directly clinically relevant.
