# Supplementary material for: Dose- and modality-specific effects of exercise on BDNF and depression in middle-aged and older adults with mild cognitive impairment: A Bayesian network meta-analysis
Source: PLoS One. 2026 Oct 7;21(10):e0358178. doi: 10.1371/journal.pone.0358178 (PMC13645019; doi:10.1371/journal.pone.0358178)
Supplement: Supplementary file 11 [file pone.0358178.s012.docx]

S1 Fig. Posterior mean estimates across overall exercise dose categories for BDNF

Notes: The boxplots present the distribution of posterior mean estimates for BDNF across different categories of overall exercise dose. The central line within each box indicates the median posterior mean, the box represents the interquartile range, and the whiskers indicate the spread of the estimates across included comparisons within each dose category. This figure was used to visually compare the central tendency and dispersion of posterior mean estimates across dose levels in the Bayesian model.
