# Supplementary material for: Dose- and modality-specific effects of exercise on BDNF and depression in middle-aged and older adults with mild cognitive impairment: A Bayesian network meta-analysis
Source: PLoS One. 2026 Oct 7;21(10):e0358178. doi: 10.1371/journal.pone.0358178 (PMC13645019; doi:10.1371/journal.pone.0358178)
Supplement: Supplementary file 12 [file pone.0358178.s013.docx]

S2 Fig. Study-specific responses on the link scale across overall exercise dose categories for BDNF

Notes: The figure displays the study-specific responses for BDNF on the link scale according to overall exercise dose categories. Each point represents one observed comparison or estimate contributed by an included study. The horizontal reference line at 0 indicates the null value on the link scale. Positive values indicate a favorable direction of effect relative to the reference condition, whereas negative values indicate the opposite direction. This plot was used to inspect the distribution, magnitude, and variability of observed responses across dose levels.
