# Supplementary material for: Dose- and modality-specific effects of exercise on BDNF and depression in middle-aged and older adults with mild cognitive impairment: A Bayesian network meta-analysis
Source: PLoS One. 2026 Oct 7;21(10):e0358178. doi: 10.1371/journal.pone.0358178 (PMC13645019; doi:10.1371/journal.pone.0358178)
Supplement: Supplementary file 13 [file pone.0358178.s014.docx]

S3 Fig. Contour-enhanced funnel plot for publication bias assessment of BDNF outcomes

Notes: The contour-enhanced funnel plot was used to assess potential publication bias or other small-study effects in studies reporting BDNF outcomes. Each dot represents one included study comparison. The vertical dashed line indicates the pooled effect estimate, and the diagonal lines indicate the pseudo 95% confidence limits around the pooled effect. The shaded regions correspond to different levels of statistical significance, allowing visual identification of whether asymmetry may be related to publication bias. Symmetry of the distribution around the pooled effect suggests a lower likelihood of substantial publication bias, whereas marked asymmetry may indicate possible reporting bias, selective publication, or heterogeneity.
