# Supplementary material for: Dose- and modality-specific effects of exercise on BDNF and depression in middle-aged and older adults with mild cognitive impairment: A Bayesian network meta-analysis
Source: PLoS One. 2026 Oct 7;21(10):e0358178. doi: 10.1371/journal.pone.0358178 (PMC13645019; doi:10.1371/journal.pone.0358178)
Supplement: Supplementary file 14 [file pone.0358178.s015.docx]

S4 Fig. Sunset funnel plot for the assessment of small-study effects and statistical power of BDNF outcomes

Notes: The sunset funnel plot was used to evaluate the evidential strength and statistical power of the studies included for BDNF outcomes. Each point represents one study comparison plotted by effect estimate and standard error. The dashed vertical line indicates the pooled effect estimate. The colored background represents statistical power regions, with darker warm colors indicating lower power and greener regions indicating higher power to detect the assumed effect size. This figure allows visual assessment of whether the available evidence is dominated by low-powered studies and whether the observed significant findings are likely to be robust. The summary statistics displayed at the bottom of the plot provide additional information on median power, proportions of studies exceeding selected power thresholds, expected effect size, observed number of significant studies, and the replication index.
