# Supplementary material for: Dose- and modality-specific effects of exercise on BDNF and depression in middle-aged and older adults with mild cognitive impairment: A Bayesian network meta-analysis
Source: PLoS One. 2026 Oct 7;21(10):e0358178. doi: 10.1371/journal.pone.0358178 (PMC13645019; doi:10.1371/journal.pone.0358178)
Supplement: Supplementary file 16 [file pone.0358178.s017.docx]

S6 Fig. Study-specific responses on the link scale across overall exercise dose categories for depressive symptoms

Notes: The figure presents study-specific responses for depressive symptoms on the link scale according to overall exercise dose categories. Each point represents one observed study comparison or estimate. The horizontal reference line at 0 indicates the null value on the link scale. Values above 0 indicate a positive response relative to the reference condition, whereas values below 0 indicate the opposite direction. This plot was used to inspect the distribution, magnitude, and heterogeneity of observed responses across dose levels.
