# Supplementary material for: Dose- and modality-specific effects of exercise on BDNF and depression in middle-aged and older adults with mild cognitive impairment: A Bayesian network meta-analysis
Source: PLoS One. 2026 Oct 7;21(10):e0358178. doi: 10.1371/journal.pone.0358178 (PMC13645019; doi:10.1371/journal.pone.0358178)
Supplement: Supplementary file 17 [file pone.0358178.s018.docx]

S7 Fig. Posterior mean estimates across modality-specific exercise dose categories for depressive symptoms

Notes: The boxplots display the distribution of posterior mean estimates for depressive symptoms across modality-specific exercise dose categories. Results are presented separately for each exercise modality, including dancing, multicomponent exercise, Tai Chi, walking, and yoga. The central line within each box indicates the median posterior mean, the box represents the interquartile range, and the whiskers indicate the spread of the estimates within each modality-specific dose category. This figure was used to compare the central tendency and variability of posterior mean estimates across different exercise modalities and dose levels in the Bayesian model.
