# Supplementary material for: Dose- and modality-specific effects of exercise on BDNF and depression in middle-aged and older adults with mild cognitive impairment: A Bayesian network meta-analysis
Source: PLoS One. 2026 Oct 7;21(10):e0358178. doi: 10.1371/journal.pone.0358178 (PMC13645019; doi:10.1371/journal.pone.0358178)
Supplement: Supplementary file 18 [file pone.0358178.s019.docx]

S8 Fig. Study-specific responses on the link scale across modality-specific exercise dose categories for depressive symptoms

Notes: The figure presents study-specific responses for depressive symptoms on the link scale across modality-specific exercise dose categories. Results are shown separately by exercise modality, including dancing, multicomponent exercise, Tai Chi, walking, and yoga. Each point represents one observed study comparison or estimate, and the connecting lines indicate responses contributed within the same modality-specific dose structure. The horizontal reference line at 0 indicates the null value on the link scale. Values above 0 indicate a positive response relative to the reference condition, whereas values below 0 indicate the opposite direction. This plot was used to inspect the magnitude, distribution, and heterogeneity of observed responses across exercise modalities and dose levels.
