# Supplementary material for: Dose- and modality-specific effects of exercise on BDNF and depression in middle-aged and older adults with mild cognitive impairment: A Bayesian network meta-analysis
Source: PLoS One. 2026 Oct 7;21(10):e0358178. doi: 10.1371/journal.pone.0358178 (PMC13645019; doi:10.1371/journal.pone.0358178)
Supplement: Supplementary file 19 [file pone.0358178.s020.docx]

S9 Fig. Contour-enhanced funnel plot for publication bias assessment of depressive symptom outcomes

Notes: The contour-enhanced funnel plot was used to assess potential publication bias or other small-study effects for studies reporting depressive symptom outcomes. Each dot represents one included study comparison. The vertical dashed line indicates the pooled effect estimate, and the diagonal lines indicate the pseudo 95% confidence limits around the pooled effect. The shaded contour regions represent different significance thresholds, which help distinguish whether funnel plot asymmetry may be related to publication bias. A roughly symmetrical distribution of studies around the pooled effect suggests a lower likelihood of substantial publication bias, whereas marked asymmetry may indicate selective publication, reporting bias, or heterogeneity.
