# Supplementary material for: Dose- and modality-specific effects of exercise on BDNF and depression in middle-aged and older adults with mild cognitive impairment: A Bayesian network meta-analysis
Source: PLoS One. 2026 Oct 7;21(10):e0358178. doi: 10.1371/journal.pone.0358178 (PMC13645019; doi:10.1371/journal.pone.0358178)
Supplement: Supplementary file 20 [file pone.0358178.s021.docx]

S10 Fig. Sunset funnel plot for the assessment of small-study effects and statistical power of depressive symptom outcomes

Notes: The sunset funnel plot was used to further evaluate the evidential strength, potential small-study effects, and statistical power of the studies included for depressive symptom outcomes. Each point represents one study comparison plotted by effect estimate and standard error. The dashed vertical line indicates the pooled effect estimate. The colored background represents statistical power regions, with warmer colors indicating lower power and greener colors indicating higher power to detect the assumed effect size. This figure provides a visual summary of whether the available evidence is dominated by low-powered studies and whether the observed significant findings are likely to be robust. The summary statistics displayed at the bottom of the plot provide additional information on median power, proportions of studies exceeding selected power thresholds, expected effect size, observed number of significant studies, and the replication index.
