# Supplementary material for: Dose- and modality-specific effects of exercise on BDNF and depression in middle-aged and older adults with mild cognitive impairment: A Bayesian network meta-analysis
Source: PLoS One. 2026 Oct 7;21(10):e0358178. doi: 10.1371/journal.pone.0358178 (PMC13645019; doi:10.1371/journal.pone.0358178)
Supplement: Supplementary file 22 [file pone.0358178.s023.docx]

S12 Fig. Functional-form sensitivity analysis for the overall exercise dose–depressive symptoms network

Notes: Based on the complete analyses rerun in R, the figure overlays the posterior mean dose–response curves from the linear, quadratic, Emax, and natural cubic spline models for the overall exercise dose–depressive symptoms network. The linear model is represented by a straight line, whereas the quadratic, Emax, and natural cubic spline models are nonlinear and therefore appear as curves. Differences in curve shape and in the location of the maximizing dose illustrate sensitivity to the assumed functional form.
