# Supplementary material for: Dose- and modality-specific effects of exercise on BDNF and depression in middle-aged and older adults with mild cognitive impairment: A Bayesian network meta-analysis
Source: PLoS One. 2026 Oct 7;21(10):e0358178. doi: 10.1371/journal.pone.0358178 (PMC13645019; doi:10.1371/journal.pone.0358178)
Supplement: Supplementary file 23 [file pone.0358178.s024.docx]

Included literature:(Allard et al., 2017; Baker et al., 2010; Bisbe et al., 2020; Buele et al., 2024; Chang et al., 2021; Damirchi et al., 2018; Eyre et al., 2017; Ho et al., 2020; Khanthong et al., 2024; Kohanpour et al., 2017; Lam et al., 2015; Lam et al., 2012; Langoni et al., 2019; Lazarou et al., 2017; Lin et al., 2025; Resende-Silva et al., 2025; Sanchez-Alcala et al., 2024; Song & Yu, 2019; Sungkarat et al., 2018; Tremont et al., 2022; Tsai et al., 2019; L. Wang et al., 2020; S. Wang et al., 2020; Yang et al., 2016; Yu et al., 2022; Zhang et al., 2024; Zhang et al., 2023; Zhu et al., 2018)

Allard, J. S., Ntekim, O., Johnson, S. P., Ngwa, J. S., Bond, V., Pinder, D., Gillum, R. F., Fungwe, T. V., Kwagyan, J., & Obisesan, T. O. (2017, Jan). APOEε4 impacts up-regulation of brain-derived neurotrophic factor after a six-month stretch and aerobic exercise intervention in mild cognitively impaired elderly African Americans: A pilot study. *Exp Gerontol, 87*(Pt A), 129-136. <https://doi.org/10.1016/j.exger.2016.11.001>

Baker, L. D., Frank, L. L., Foster-Schubert, K., Green, P. S., Wilkinson, C. W., McTiernan, A., Plymate, S. R., Fishel, M. A., Watson, G. S., Cholerton, B. A., Duncan, G. E., Mehta, P. D., & Craft, S. (2010, Jan). Effects of aerobic exercise on mild cognitive impairment: a controlled trial. *Arch Neurol, 67*(1), 71-79. <https://doi.org/10.1001/archneurol.2009.307>

Bisbe, M., Fuente-Vidal, A., López, E., Moreno, M., Naya, M., de Benetti, C., Milà, R., Bruna, O., Boada, M., & Alegret, M. (2020). Comparative Cognitive Effects of Choreographed Exercise and Multimodal Physical Therapy in Older Adults with Amnestic Mild Cognitive Impairment: Randomized Clinical Trial. *Journal of Alzheimer’s Disease, 73*(2), 769-783. <https://doi.org/10.3233/jad-190552>

Buele, J., Avilés-Castillo, F., Soto, C. D., Varela-Aldás, J., & Palacios-Navarro, G. (2024, Aug 1). Effects of a dual intervention (motor and virtual reality-based cognitive) on cognition in patients with mild cognitive impairment: a single-blind, randomized controlled trial. *Journal of Neuroengineering and Rehabilitation, 21*(1), Article 130. <https://doi.org/10.1186/s12984-024-01422-w>

Chang, J., Zhu, W., Zhang, J., Yong, L., Yang, M., Wang, J., & Yan, J. (2021). The Effect of Chinese Square Dance Exercise on Cognitive Function in Older Women With Mild Cognitive Impairment: The Mediating Effect of Mood Status and Quality of Life. *Front Psychiatry, 12*, 711079. <https://doi.org/10.3389/fpsyt.2021.711079>

Damirchi, A., Hosseini, F., & Babaei, P. (2018, Feb). Mental Training Enhances Cognitive Function and BDNF More Than Either Physical or Combined Training in Elderly Women With MCI: A Small-Scale Study. *Am J Alzheimers Dis Other Demen, 33*(1), 20-29. <https://doi.org/10.1177/1533317517727068>

Eyre, H. A., Siddarth, P., Acevedo, B., Van Dyk, K., Paholpak, P., Ercoli, L., St Cyr, N., Yang, H., Khalsa, D. S., & Lavretsky, H. (2017, Apr). A randomized controlled trial of Kundalini yoga in mild cognitive impairment. *Int Psychogeriatr, 29*(4), 557-567. <https://doi.org/10.1017/s1041610216002155>

Ho, R. T. H., Fong, T. C. T., Chan, W. C., Kwan, J. S. K., Chiu, P. K. C., Yau, J. C. Y., & Lam, L. C. W. (2020, Feb 14). Psychophysiological Effects of Dance Movement Therapy and Physical Exercise on Older Adults With Mild Dementia: A Randomized Controlled Trial. *J Gerontol B Psychol Sci Soc Sci, 75*(3), 560-570. <https://doi.org/10.1093/geronb/gby145>

Khanthong, P., Sriyakul, K., Dechakhamphu, A., Krajarng, A., Kamalashiran, C., Jayathavaj, V., & Tungsukruthai, P. (2024, Aug). A randomized controlled trial on the effects of traditional Thai mind-body exercise (Ruesi Dadton) on biomarkers in mild cognitive impairment. *European Journal of Physical and Rehabilitation Medicine, 60*(4), 604-610. <https://doi.org/10.23736/s1973-9087.24.08015-8>

Kohanpour, M.-A., Peeri, M., & Azarbayjani, M. A. (2017, 04/01). The effects of aerobic exercise with lavender essence use on cognitive state and serum brain-derived neurotrophic factor levels in elderly with mild cognitive impairment. *Journal of Herbmed Pharmacology, 6*, 80-84.

Lam, L. C., Chan, W. C., Leung, T., Fung, A. W., & Leung, E. M. (2015). Would older adults with mild cognitive impairment adhere to and benefit from a structured lifestyle activity intervention to enhance cognition?: a cluster randomized controlled trial. *PLoS One, 10*(3), e0118173. <https://doi.org/10.1371/journal.pone.0118173>

Lam, L. C., Chau, R. C., Wong, B. M., Fung, A. W., Tam, C. W., Leung, G. T., Kwok, T. C., Leung, T. Y., Ng, S. P., & Chan, W. M. (2012, Jul). A 1-year randomized controlled trial comparing mind body exercise (Tai Chi) with stretching and toning exercise on cognitive function in older Chinese adults at risk of cognitive decline. *J Am Med Dir Assoc, 13*(6), 568.e515-520. <https://doi.org/10.1016/j.jamda.2012.03.008>

Langoni, C. D. S., Resende, T. L., Barcellos, A. B., Cecchele, B., da Rosa, J. N., Knob, M. S., Silva, T. D. N., Diogo, T. S., da Silva, I. G., & Schwanke, C. H. A. (2019, Mar). The effect of group exercises on balance, mobility, and depressive symptoms in older adults with mild cognitive impairment: a randomized controlled trial. *Clin Rehabil, 33*(3), 439-449. <https://doi.org/10.1177/0269215518815218>

Lazarou, I., Parastatidis, T., Tsolaki, A., Gkioka, M., Karakostas, A., Douka, S., & Tsolaki, M. (2017). International Ballroom Dancing Against Neurodegeneration: A Randomized Controlled Trial in Greek Community-Dwelling Elders With Mild Cognitive impairment. *American Journal of Alzheimer's Disease & Other Dementias®, 32*(8), 489-499. <https://doi.org/10.1177/1533317517725813>

Lin, L., He, Y. X., Wen, Q., Liu, J. Y., Dai, Y., Fei, Y. Z., Li, H., Li, C. Q., & Zhou, H. (2025, Apr 2). Evaluation of the efficacy of Tai Chi on the cognitive function of patients with mild cognitive dysfunction and research on its mechanism. *Frontiers in Aging Neuroscience, 17*, Article 1435996. <https://doi.org/10.3389/fnagi.2025.1435996>

Resende-Silva, S., de Resende-Neto, A. G., Vasconcelos, A. B. S., Pereira-Monteiro, M. R., Pantoja-Cardoso, A., Santos, L. E. S., Lima, N. B. C., Santos, J., Aragao-Santos, J. C., Schimieguel, D. M., dos Santos, J. R., & Da Silva-Grigoletto, M. E. (2025, Sep 11). Functional training improves cognitive function, functional fitness, and BDNF levels in older women with mild cognitive impairment: a randomized controlled trial. *Frontiers in Physiology, 16*, Article 1638590. <https://doi.org/10.3389/fphys.2025.1638590>

Sanchez-Alcala, M., Aibar-Almazán, A., Hita-Contreras, F., Castellote-Caballero, Y., Carcelén-Fraile, M. D., Infante-Guedes, A., & González-Martín, A. M. (2024, Aug). Effects of Dance-Based Aerobic Training on Mental Health and Quality of Life in Older Adults with Mild Cognitive Impairment. *Journal of Personalized Medicine, 14*(8), Article 844. <https://doi.org/10.3390/jpm14080844>

Song, D., & Yu, D. S. F. (2019, May). Effects of a moderate-intensity aerobic exercise programme on the cognitive function and quality of life of community-dwelling elderly people with mild cognitive impairment: A randomised controlled trial. *Int J Nurs Stud, 93*, 97-105. <https://doi.org/10.1016/j.ijnurstu.2019.02.019>

Sungkarat, S., Boripuntakul, S., Kumfu, S., Lord, S. R., & Chattipakorn, N. (2018, Feb). Tai Chi Improves Cognition and Plasma BDNF in Older Adults With Mild Cognitive Impairment: A Randomized Controlled Trial. *Neurorehabil Neural Repair, 32*(2), 142-149. <https://doi.org/10.1177/1545968317753682>

Tremont, G., Davis, J., Ott, B. R., Uebelacker, L., Kenney, L., Gillette, T., Britton, K., & Sanborn, V. (2022, Mar). Feasibility of a Yoga Intervention for Individuals with Mild Cognitive Impairment: A Randomized Controlled Trial. *J Integr Complement Med, 28*(3), 250-260. <https://doi.org/10.1089/jicm.2021.0204>

Tsai, C. L., Pai, M. C., Ukropec, J., & Ukropcová, B. (2019). Distinctive Effects of Aerobic and Resistance Exercise Modes on Neurocognitive and Biochemical Changes in Individuals with Mild Cognitive Impairment. *Curr Alzheimer Res, 16*(4), 316-332. <https://doi.org/10.2174/1567205016666190228125429>

Wang, L., Wu, B., Tao, H., Chai, N., Zhao, X., Zhen, X., & Zhou, X. (2020, Oct). Effects and mediating mechanisms of a structured limbs-exercise program on general cognitive function in older adults with mild cognitive impairment: A randomized controlled trial. *Int J Nurs Stud, 110*, 103706. <https://doi.org/10.1016/j.ijnurstu.2020.103706>

Wang, S., Yin, H., Meng, X., Shang, B., Meng, Q., Zheng, L., Wang, L., & Chen, L. (2020, May-Jun). Effects of Chinese square dancing on older adults with mild cognitive impairment. *Geriatr Nurs, 41*(3), 290-296. <https://doi.org/10.1016/j.gerinurse.2019.10.009>

Yang, H., Leaver, A. M., Siddarth, P., Paholpak, P., Ercoli, L., St. Cyr, N. M., Eyre, H. A., Narr, K. L., Khalsa, D. S., & Lavretsky, H. (2016, 2016-November-21). Neurochemical and Neuroanatomical Plasticity Following Memory Training and Yoga Interventions in Older Adults with Mild Cognitive Impairment [Original Research]. *Frontiers in Aging Neuroscience, Volume 8 - 2016*. <https://doi.org/10.3389/fnagi.2016.00277>

Yu, D. J., Yu, A. P., Bernal, J. D. K., Fong, D. Y., Chan, D. K. C., Cheng, C. P., & Siu, P. M. (2022, 2022-September-19). Effects of exercise intensity and frequency on improving cognitive performance in middle-aged and older adults with mild cognitive impairment: A pilot randomized controlled trial on the minimum physical activity recommendation from WHO [Original Research]. *Frontiers in Physiology, Volume 13 - 2022*. <https://doi.org/10.3389/fphys.2022.1021428>

Zhang, J., Yang, C., Pan, Y. J., & Wang, L. (2024, Mar-Apr). Effects of Multicomponent Exercise on Community-Dwelling Older Adults With Mild Cognitive Impairment. *Research in Gerontological Nursing, 17*(2), 65-79. <https://doi.org/10.3928/19404921-20240112-02>

Zhang, Q., Zhu, M. L., Huang, L. L., Zhu, M., Liu, X. L., Zhou, P., & Meng, T. (2023, Jul). A Study on the Effect of Traditional Chinese Exercise Combined With Rhythm Training on the Intervention of Older Adults With Mild Cognitive Impairment. *American Journal of Alzheimers Disease and Other Dementias, 38*, Article 15333175231190626. <https://doi.org/10.1177/15333175231190626>

Zhu, Y., Wu, H., Qi, M., Wang, S., Zhang, Q., Zhou, L., Wang, S., Wang, W., Wu, T., Xiao, M., Yang, S., Chen, H., Zhang, L., Zhang, K. C., Ma, J., & Wang, T. (2018). Effects of a specially designed aerobic dance routine on mild cognitive impairment. *Clin Interv Aging, 13*, 1691-1700. <https://doi.org/10.2147/cia.S163067>
